# TAB2 deficiency induces dilated cardiomyopathy by promoting RIPK1-dependent apoptosis and necroptosis

**DOI:** 10.1172/JCI152297

**Published:** 2022-02-15

**Authors:** Haifeng Yin, Xiaoyun Guo, Yi Chen, Yachang Zeng, Xiaoliang Mo, Siqi Hong, Hui He, Jing Li, Rachel Steinmetz, Qinghang Liu

**Affiliations:** 1 Department of Physiology and Biophysics, University of Washington, Seattle, Washington, USA.

**Keywords:** Cardiology, Cell Biology, Apoptosis, Cardiovascular disease, Molecular biology

## Abstract

Mutations in TGF-β–activated kinase 1 binding protein 2 (*TAB2*) have been implicated in the pathogenesis of dilated cardiomyopathy and/or congenital heart disease in humans, but the underlying mechanisms are currently unknown. Here, we identified an indispensable role for TAB2 in regulating myocardial homeostasis and remodeling by suppressing receptor-interacting protein kinase 1 (RIPK1) activation and RIPK1-dependent apoptosis and necroptosis. Cardiomyocyte-specific deletion of *Tab2* in mice triggered dilated cardiomyopathy with massive apoptotic and necroptotic cell death. Moreover, *Tab2*-deficient mice were also predisposed to myocardial injury and adverse remodeling after pathological stress. In cardiomyocytes, deletion of TAB2 but not its close homolog TAB3 promoted TNF-α–induced apoptosis and necroptosis, which was rescued by forced activation of TAK1 or inhibition of RIPK1 kinase activity. Mechanistically, TAB2 critically mediates RIPK1 phosphorylation at Ser321 via a TAK1-dependent mechanism, which prevents RIPK1 kinase activation and the formation of RIPK1-FADD-caspase-8 apoptotic complex or RIPK1-RIPK3 necroptotic complex. Strikingly, genetic inactivation of RIPK1 with *Ripk1*-K45A knockin effectively rescued cardiac remodeling and dysfunction in *Tab2*-deficient mice. Together, these data demonstrated that TAB2 is a key regulator of myocardial homeostasis and remodeling by suppressing RIPK1-dependent apoptosis and necroptosis. Our results also suggest that targeting RIPK1-mediated cell death signaling may represent a promising therapeutic strategy for TAB2 deficiency–induced dilated cardiomyopathy.

## Graphical abstract

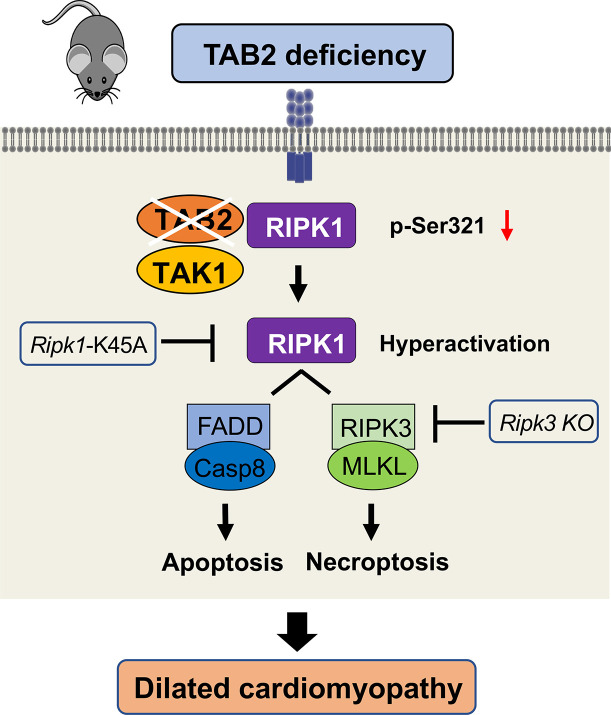

## Introduction

Loss of cardiomyocytes by apoptotic and necrotic death is a crucial event underlying pathological cardiac remodeling and heart failure (1, 2). Similar to apoptosis, emerging evidence indicates that necrosis can also occur in a highly regulated and genetically controlled manner, termed regulated necrosis. A number of regulated necrosis pathways have recently been identified, including necroptosis, ferroptosis, pyroptosis, mitochondria-mediated necrosis, and other regulated necrotic processes (1). Necroptosis is a caspase-independent, highly regulated necrotic cell death modality, which has recently been implicated in ischemic cardiac injury and pathological remodeling (3–5). Necroptosis is executed through the induction of receptor-interacting protein kinase 1 and 3 (RIPK1-RIPK3) necrosome, phosphorylation and oligomerization of mixed lineage kinase domain-like (MLKL), and plasma membrane disruption (6–9).

Like apoptosis, necroptosis is induced by specific death receptors, such as TNF receptor 1 (TNFR1), among other modules. Stimulation of TNFR1 by TNF-α can trigger a variety of cellular responses, including cell survival, apoptosis, and necroptosis, depending on the cellular context. Under normal conditions, ligation of TNFR1 triggers the assembly of a plasma membrane bound signaling complex, termed complex I, consisting of TNFR-associated protein with death domain (TRADD), TNFR-associated protein 2 (TRAF2), cellular inhibitor of apoptosis protein 1 and 2 (cIAP1 and cIAP2), and RIPK1 (10). Within complex I, RIPK1 is Lys63-ubiquitinated by ubiquitin ligases TRAF2 and cIAP1/2, which then recruits and activates TGF-β–activated kinase 1 (TAK1) and the IκB kinase (IKK) complex, leading to the activation of NF-κB and transcription of prosurvival genes. NF-κB–mediated induction of prosurvival molecules is a well-defined cell death checkpoint in the TNFR1 pathway (11). Under apoptosis-inducing conditions, such as inhibition of NF-κB, the TNFR1 complex internalizes and converts to a cell death–inducing complex, termed complex II, consisting of TRADD, Fas-associated protein with death domain (FADD), and caspase-8 (10). Recent studies identified another cell death checkpoint, which is controlled by RIPK1 kinase activity. Indeed, it has been shown that inhibition of TAK1, NEMO, IKK-α, IKK-β, or cIAP1/2 promotes RIPK1 activation and sensitizes cells to RIPK1-dependent apoptosis or necroptosis independently of the NF-κB pathway (12–14). RIPK1 kinase activation induces cell death either by promoting the assembly of RIPK1-FADD-caspase-8 apoptotic complex (13, 15, 16) or RIPK1-RIPK3-MLKL necroptotic complex (17). RIPK1 kinase activity is tightly regulated by posttranslational modifications, such as ubiquitination and phosphorylation (12, 16, 18–20). However, the molecular signaling events that control the RIPK1 kinase-dependent cell death checkpoint remain elusive.

TGF-β–activated kinase 1 binding protein 2 (TAB2) is an adaptor protein linking signals from TNFR1 and other receptors to the TAK1 signaling complex by binding to Lys63-linked polyubiquitin chains (21). TAK1 has recently been identified as a key regulator of apoptosis and necroptosis through NF-κB–dependent and –independent mechanisms (14, 16). TAK1 forms a complex with TAB1 and TAB2 or TAB3, where TAB1 functions as an activator of TAK1 by promoting TAK1 oligomerization and autophosphorylation (22). TAB2 and its homolog TAB3 are shown to play redundant roles in TAK1 activation and downstream signaling (23, 24). Intriguingly, TAB2 also mediates TAK1 deactivation by recruiting PP6 to the TAK1 complex (25, 26). Therefore, TAB2 may function as a TAK1 activator or suppressor depending on cell types or cellular contexts. It has been controversial as to whether TAB2 plays a role in apoptosis, necroptosis, or both. For example, Sanjo et al. showed that deletion of TAB2 in mouse embryonic fibroblasts (MEFs) had no effects on TNF-α–induced cell death (23). In contrast, TAB2*^–/–^* dermal fibroblasts displayed increased sensitivity to TNF-α–induced necroptosis but not apoptosis (26). Moreover, TAB2-deficient hepatocytes showed increased apoptotic cell death and caspase-3 activation after LPS stimulation (27). Therefore, the role of TAB2 in apoptotic and necroptotic cell death has not been unequivocally established, and the mechanisms by which TAB2 regulates cell death signaling remain largely unknown.

Genetic deletion of *Tab2* in mice led to embryonic lethality with massive cell death in the fetal liver (23). In contrast, *Tab3^–/–^* mice showed no overt basal phenotype (28). These results suggest an indispensable and nonredundant role for TAB2, rather than TAB3, in tissue survival and hemostasis. The human *TAB2* gene is mapped to chromosome 6q25.1. Haploinsufficiency of *TAB2* caused by microdeletions of chromosome 6q25.1 in humans is associated with congenital heart defects (CHDs) and/or cardiomyopathy (29–35). CHDs and cardiomyopathy are also reported in patients with missense, nonsense, and small insertion or deletion mutations within *TAB2* (32, 33, 34). Of note, some patients developed cardiomyopathy (mostly dilated cardiomyopathy) in the absence of CHDs, and cardiomyopathy can also occur later in life in those who survived CHDs (30, 31, 33). Most of the pathological effects are caused by loss of function of the *TAB2* allele, although gain-of-function mutation of *TAB2* has also been reported (35). The molecular mechanisms underlying cardiac abnormalities caused by TAB2 mutations remain elusive.

Here, we identified an essential role for TAB2 in myocardial homeostasis and survival by suppressing apoptosis and necroptosis primarily through a RIPK1-dependent mechanism. By generating cardiomyocyte-specific *Tab2*-KO mouse models, we showed that genetic ablation of TAB2 in the adult heart promoted apoptotic and necroptotic cell death, leading to dilated cardiomyopathy and heart failure. We also provide evidence that genetic inhibition of RIPK1 kinase activity effectively rescued cardiac dysfunction and pathology associated with TAB2 deficiency in vivo, revealing a TAB2-mediated, RIPK1-dependent apoptotic and necroptotic signaling pathway in regulating myocardial homeostasis and remodeling.

## Results

### Loss of TAB2 in the adult heart induces dilated cardiomyopathy and heart failure.

**Figure 1:**
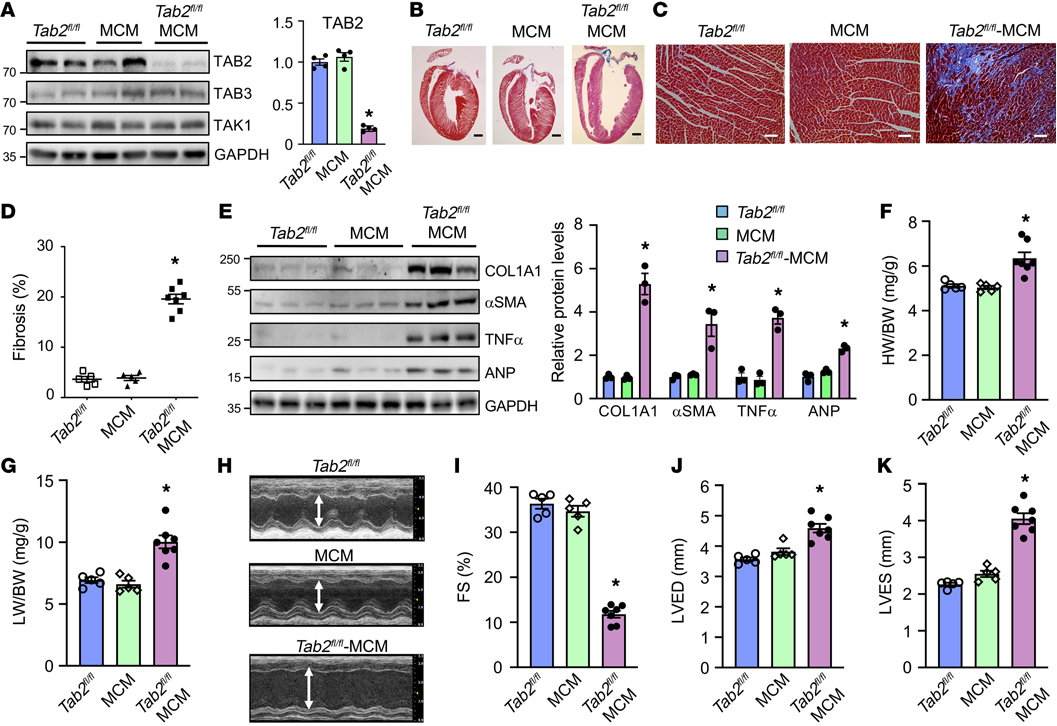
Cardiomyocyte-specific ablation of TAB2 leads to dilated cardiomyopathy in mice. (**A**) Western blotting for the indicated proteins in ventricular extracts from *Tab2^fl/fl^*, MerCreMer (MCM), and *Tab2^fl/fl^*-MCM mice 2 weeks after treatment with tamoxifen as described in Methods. TAB2 protein level was normalized for the internal control GAPDH and expressed as fold change. *P < 0.05 versus *Tab2^fl/fl^* or MCM. *n =* 4. (**B** and **C**) Masson’s trichrome–stained, paraffin-embedded cardiac sections from mice as described in **A**. Scale bars: 1 mm in **B** and 50 μm in **C**. (**D**) Myocardial fibrosis quantified with MetaMorph software. *P < 0.05 versus *Tab2^fl/fl^* or MCM. *n =* 5–7. (**E**) Western blot (left) and quantification (right) of the indicated proteins normalized to GAPDH in cardiac extracts from mice indicated in **A**. (**F**) Heart weight to body weight ratio (HW/BW) of mice of the indicated genotypes. *P < 0.05 versus *Tab2^fl/fl^* or MCM. (**G**) Lung weight to body weight ratio (LW/BW) of mice of the indicated genotypes. *P < 0.05 versus *Tab2^fl/fl^* or MCM. (**H**) Representative echocardiographic M-mode images from mice indicated in **A**. The vertical white arrowed lines indicate left ventricular dimension in end-diastole (LVED). (**I**–**K**) Echocardiographic assessment of fractional shortening (FS) and left ventricular dimension in end-diastole (LVED) and end-systole (LVES) in mice of the indicated genotypes. *P < 0.05 versus *Tab2^fl/fl^* or MCM. *n =* 5–7. Statistical analysis was performed using 1-way ANOVA with Tukey’s post hoc test.

To investigate the role of TAB2 in the adult heart, we used a tamoxifen-inducible Cre-mediated recombination system for acute deletion of *Tab2* in cardiomyocytes. Mice homozygous for the *Tab2*-*loxp* targeted allele (*Tab2^fl/fl^*; ref. 23) were crossed with αMHC-MerCreMer (MCM) transgenic mice (36), which in the absence of tamoxifen were overtly normal with no detectable phenotype at baseline (data not shown). Western blot analysis showed that TAB2 was efficiently deleted (>90%) from the hearts of *Tab2^fl/fl^*-MCM mice but not *Tab2^fl/fl^* or MCM control mice after tamoxifen treatment (Figure 1A). TAB3 or TAK1 expression was not altered (Figure 1A). Strikingly, after 2 weeks of tamoxifen administration, *Tab2^fl/fl^*-MCM mice rapidly developed severe ventricular dilation with high levels of myocardial fibrosis (Figure 1, B–D). Moreover, the protein levels of fibrotic genes (collagen type 1 and α-smooth muscle actin), as well as atrial natriuretic peptide (ANP) and TNF-α were substantially elevated in TAB2-deficient hearts (Figure 1E). *Tab2^fl/fl^*-MCM mice also displayed cardiac hypertrophy and pulmonary congestion as assessed by heart weight to body weight and lung weight to body weight ratios (Figure 1, F and G), suggesting that TAB2 deficiency induced pathological cardiac remodeling and congestive heart failure. Echocardiographic analysis showed severe ventricular dilation and contractile dysfunction in TAB2-deficient mice, as indicated by decreased fractional shortening (FS) and increased left ventricular dimensions in end-diastole and end-systole (LVED and LEVS) (Figure 1, H–K). Moreover, *Tab2^fl/fl^*-MCM mice showed a progressive deterioration of cardiac function, and most of these mice died 6 weeks after tamoxifen administration (Supplemental Figure 1; supplemental material available online with this article; https://doi.org/10.1172/JCI152297DS1). Therefore, acute deletion of *Tab2* in the adult heart induced cardiac remodeling and heart failure, recapitulating the phenotype of dilated cardiomyopathy in humans with *TAB2* gene mutations (30–33).

### Ablation of TAB2 induces apoptotic and necroptotic cell death in the myocardium.

**Figure 2:**
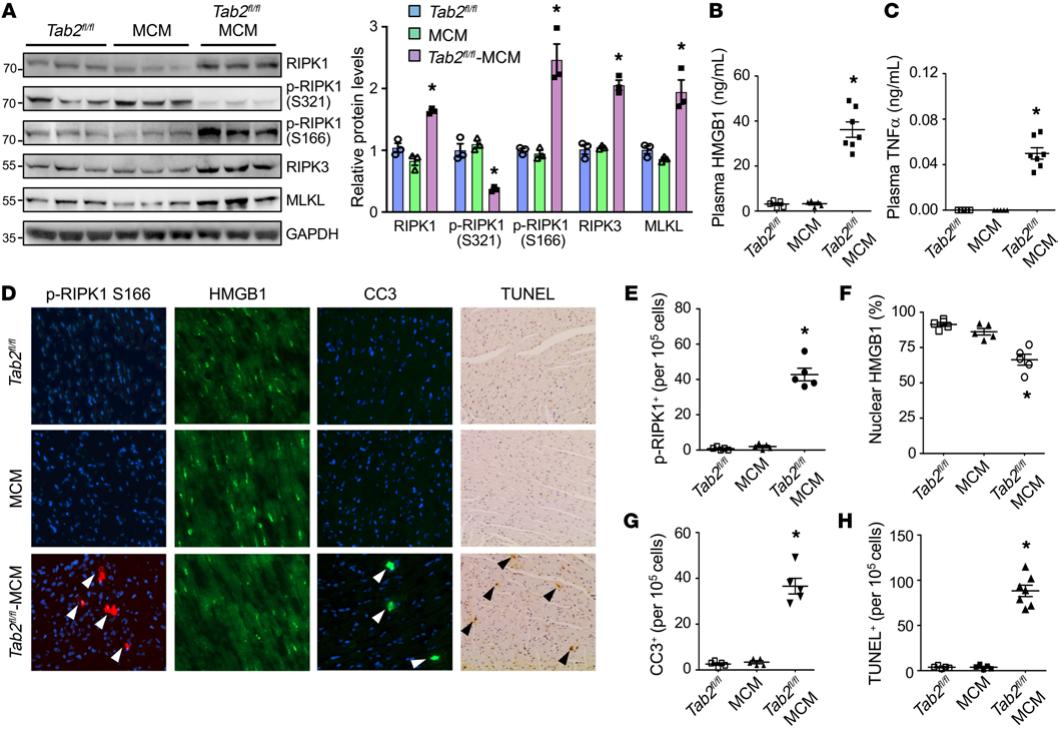
Loss of TAB2 promotes myocardial apoptosis and necroptosis. (**A**) Western blot (left) and quantification (right) of the indicated proteins normalized to GAPDH in cardiac extracts from *Tab2^fl/fl^*, MCM, and *Tab2^fl/fl^*-MCM mice 2 weeks after tamoxifen treatment. (**B** and **C**) Plasma HMGB1 and TNF-α levels from mice indicated in **A**. *P < 0.05 versus *Tab2^fl/fl^* or MCM. *n =* 5–7. (**D**) Immunofluorescence staining with the indicated antibodies as well as TUNEL assay in cardiac sections from mice of the indicated genotypes. (**E**–**H**) Quantification of phospho-RIPK1 Ser166, nuclear HMGB1, cleaved caspase-3 (CC3), and TUNEL-positive cells. *P < 0.05 versus *Tab2^fl/fl^* or MCM. *n =* 5–7. Statistical analysis was performed using 1-way ANOVA with Tukey’s post hoc test.

Next, we assessed whether apoptotic or necrotic cell death contributes to the pathological cardiac phenotype of *Tab2* deficiency. Intriguingly, protein levels of RIPK1, RIPK3, and MLKL, key mediators of necroptosis, were significantly increased in the heart extracts of *Tab2^fl/fl^*-MCM mice compared with littermate controls (Figure 2A). The level of phospho-RIPK1 at Ser 166, an established marker for RIPK1 kinase activation (37), was greatly enhanced, whereas phospho-RIPK1 at Ser321, which has recently been shown to suppress RIPK1 kinase activation (16), was markedly decreased in TAB2-deficient hearts (Figure 2A). Moreover, a marked increase in the plasma levels of HMGB1 (Figure 2B), a biomarker for necrotic cell death and myocardial injury (5, 38, 39), was also detected in TAB2-deficient mice, along with elevated TNF-α levels in the plasma (Figure 2C). Immunofluorescent staining of cardiac sections also revealed enhanced RIPK1 phosphorylation at Ser166 in TAB2-deficient hearts, indicating RIPK1 kinase activation (Figure 2, D and E). Loss of nuclear HMGB1, an indicator of membrane disruption and necrosis (5, 38,^39^), was also readily detectable (Figure 2, D and F). Of note, TAB2-deficient mice also exhibited a marked increase in cleaved caspase-3 and TUNEL-positive cells (Figure 2, D, G, and H), indicating the induction of apoptosis. Together, these data indicate that loss of TAB2 promotes apoptotic and necroptotic cell death in the myocardium, suggesting an essential role of TAB2 in myocardial survival and homeostasis.

### TAB2 deficiency leads to exacerbated cardiac remodeling and dysfunction after pathological stress.

**Figure 3:**
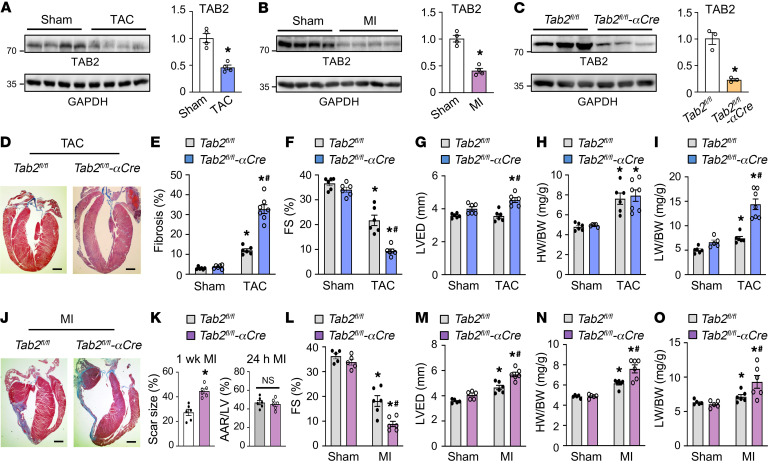
TAB2-deficient mice are predisposed to adverse cardiac remodeling and dysfunction after pathological stress. (**A** and **B**) Western blot and quantification of cardiac TAB2 levels normalized to GAPDH in WT mice subjected to TAC versus sham (**A**) or MI versus sham (**B**) for 2 weeks. *P < 0.05 versus sham. *n =* 4. (**C**) Western blot and quantification of cardiac TAB2 levels in *Tab2^fl/fl^* and *Tab2^fl/fl^*-αMHC-Cre mice at 2 months of age. *P < 0.05 versus *Tab2^fl/fl^*. *n =* 3. (**D**) Masson’s trichrome–stained cardiac sections from mice of the indicated genotypes 1 week after TAC. (**E**) Quantification of cardiac fibrosis. *P < 0.05 versus sham. ^#^*P* < 0.05 versus *Tab2^fl/fl^* TAC. *n =* 6–7. (**F** and **G**) Echocardiographic measurement of FS and LVED. *P < 0.05 versus sham. ^#^*P* < 0.05 versus *Tab2^fl/fl^* TAC. (**H**) HW/BW ratio of the indicated mice. *P < 0.05 versus sham. (**I**) LW/BW ratio of the indicated mice. *P < 0.05 versus sham. ^#^*P*< 0.05 versus *Tab2^fl/fl^* TAC. (**J**) Masson’s trichrome–stained cardiac sections from the indicated mice 1 week after MI. (**K**) Infarct scar size of the indicated mice subjected to MI for 1 week (left). Initial area at risk (AAR) normalized to area of the left ventricle (LV) was measured 24 hours after MI. *P < 0.05 versus *Tab2^fl/fl^*. *n =* 6. (**L** and **M**) FS and LVED measured by echocardiography. *P < 0.05 versus sham. ^#^*P* < 0.05 versus *Tab2^fl/fl^* MI. *n =* 5–6. (**N** and **O**) HW/BW and LW/BW ratios. *P < 0.05 versus sham. ^#^*P* < 0.05 versus *Tab2^fl/fl^* MI. *n =* 5–6. Data were analyzed by Student’s *t* test for comparisons between 2 groups and by 2-way ANOVA with Tukey’s multiple-comparison test for consideration of genotypes and treatments.

We further examined whether TAB2 plays a role in regulating myocardial remodeling and heart failure propensity after pathological stimulation. We first measured TAB2 protein expression in the heart subjected to transverse aortic constriction (TAC) or myocardial infarction (MI). Myocardial TAB2 protein expression was markedly decreased after TAC and MI injury (Figure 3, A and B, and Supplemental Figure 2, E and F), suggesting a potential role of TAB2 in cardiac response to pathological stress. We further determined that TAB2 was markedly downregulated in cardiomyocytes isolated from mouse hearts after MI and TAC, whereas no significant change was detected in noncardiomyocytes (Supplemental Figure 2, A–D). Given that acute deletion of TAB2 in the heart induced severe cardiac remodeling and failure, we generated another cardiomyocyte-specific *Tab2*-KO mouse model by crossing *Tab2^fl/fl^* mice with αMHC-Cre mice (40). Here, TAB2 was again efficiently deleted in the heart from *Tab2^fl/fl^*-αMHC-Cre mice (Figure 3C). Echocardiographic analysis showed that *Tab2^fl/fl^*-αMHC-Cre mice were overtly normal at baseline within 2 months after birth (Supplemental Figure 3). However, these mice slowly developed contractile dysfunction or ventricular dilation starting at 3 months of age (Supplemental Figure 3). In addition, inactivation of RIPK1 with *Ripk1*-K45A knockin largely rescued cardiac dysfunction and remodeling in *Tab2^fl/fl^*-αMHC-Cre mice (Supplemental Figure 4). To evaluate the role of TAB2 in TAC- or MI-induced pathological remodeling and heart failure, here we used young *Tab2^fl/fl^*-αMHC-Cre mice (6 weeks of age), which displayed no detectable cardiac pathology at baseline. *Tab2^fl/fl^*-αMHC-Cre mice showed exacerbated cardiac remodeling and ventricular dilation after TAC, with a greater loss in cardiac contractile performance, greater cardiac fibrosis, and more prominent lung edema compared with *Tab2^fl/fl^* controls (Figure 3, D–G, and I). No significant difference in TAC-induced cardiac hypertrophy was detected between these 2 groups (Figure 3H). These results indicate that TAB2-deficient mice mainly developed dilated cardiomyopathy after TAC stimulation. The blunted hypertrophic response is possibly caused by impaired hypertrophic signaling in the absence of TAB2 (41). Moreover, a greater propensity for cardiac remodeling and dysfunction was also observed in *Tab2^fl/fl^*-αMHC-Cre mice subjected to MI injury, showing increased infarct expansion, exacerbated contractile dysfunction, enhanced ventricular dilation, increased heart size, and more severe lung edema compared with *Tab2^fl/fl^* mice (Figure 3, J–O). Moreover, *Tab2^fl/fl^*-αMHC-Cre mice also exhibited a marked increase in TUNEL-positive cells as well as phospho-RIPK1 Ser166 (Supplemental Figure 5). Taken together, loss of TAB2 promotes cardiac remodeling and dysfunction during disease stimulation, suggesting a critical cardioprotective role for TAB2 in response to pathological stress.

### TAB2 but not TAB3 is a key regulator of RIPK1-dependent apoptosis and necroptosis.

**Figure 4:**
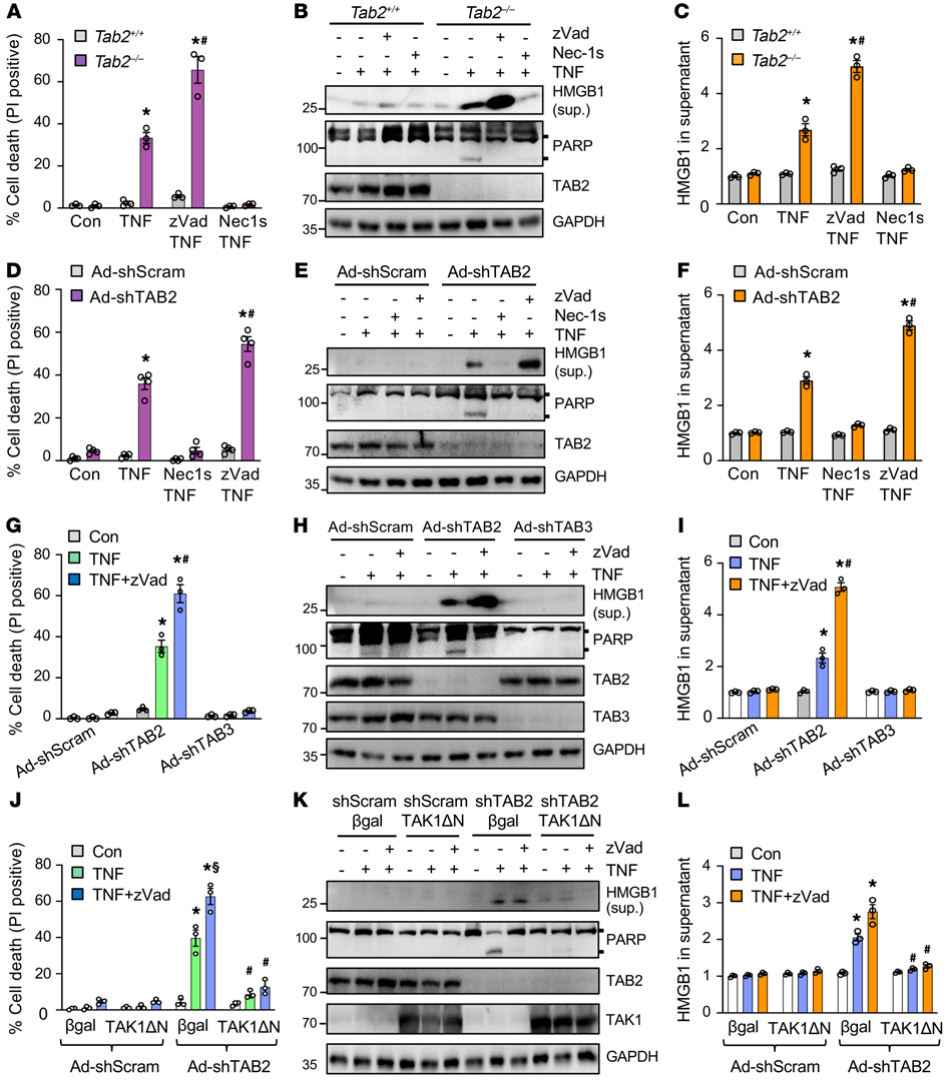
TAB2 but not TAB3 is a key regulator of apoptotic and necroptotic cell death in cardiomyocytes. (**A**) Cell death assessed by propidium iodide (PI) staining of *Tab2^+/+^* and *Tab2^–/–^* MEFs treated with 10 ng/ mL TNF-α or vehicle control for 6 hours in the presence or absence of necrostatin-1s (Nec-1s; RIPK1 inhibitor) or zVad-fmk (zVad; pan-caspase inhibitor). *P < 0.01 versus control; ^#^*P* < 0.05 versus *Tab2^–/–^* TNF. *n =* 3. (**B**) Western blotting for the indicated proteins. Sup., culture supernatant. (**C**) HMGB1 in cell culture supernatant. *P < 0.05 versus control; ^#^*P*< 0.05 versus *Tab2^–/–^* TNF. *n =* 3. (**D**) Cell death in neonatal cardiomyocytes infected with an adenovirus encoding TAB2 shRNA (shTAB2) or a scrambled sequence (shScram), and treated as indicated. *P < 0.01 versus control; ^#^*P* < 0.05 versus shTAB2 TNF. *n =* 4. (**E**) Western blotting for the indicated proteins. (**F**) HMGB1 in cell culture supernatant. *P < 0.05 versus control; ^#^*P* < 0.05 versus shTAB2 TNF. *n =* 3. (**G**) Cell death in neonatal cardiomyocytes treated as indicated. *P < 0.01 versus control; ^#^*P* < 0.05 versus shTAB2 TNF. *n =* 3. (**H**) Western blotting for the indicated proteins. (**I**) HMGB1 in supernatant. *P < 0.05 versus control; ^#^*P* < 0.05 versus shTAB2 TNF. *n =* 3. (**J**) Cell death in neonatal cardiomyocytes treated as indicated. TAKΔN indicates the constitutively active TAK1 mutant. *P < 0.01 versus control; ^#^*P* < 0.05 versus β-gal in the corresponding group; ^§^*P* < 0.05 versus Ad-shTAB2 β-gal TNF. *n =* 3. (**K**) Western blotting for the indicated proteins. (**L**) HMGB1 in supernatant. *P < 0.05 versus control; ^#^*P* < 0.05 versus β-gal in the corresponding group. *n =* 3. Statistical analysis was performed using 2-way ANOVA with Tukey’s multiple-comparison test.

Based on the data above from our *Tab2*-deficient mice, we hypothesize that *Tab2* deficiency triggers adverse cardiac remodeling and heart failure by promoting apoptosis and/or necroptosis. We first assessed whether ablation of TAB2 is sufficient to promote TNF-α–induced cell death in *Tab2*-deficient MEFs. Compared with WT MEFs, *Tab2*-deficient MEFs were highly sensitive to TNF-α stimulation, leading to rapid cell death with increased propidium iodide uptake (Figure 4A). This effect was abolished by cotreatment with the specific RIPK1 inhibitor necrostatin-1s (Nec-1s; ref. 37), but not by the pan-caspase inhibitor zVad-fmk (zVad), suggesting the induction of necroptosis (Figure 4A). Moreover, HMGB1, a biomarker for necrosis, was readily detectable in the culture supernatants of *Tab2*-deficient MEFs after TNF-α stimulation, which was further increased in the presence of zVad (Figure 4, B and C), consistent with the notion that caspase inhibition promotes necroptosis (42). Intriguingly, deletion of TAB2 also enhanced TNF-α–induced poly (ADP-ribose) polymerase (PARP) cleavage, which was reversed by both Nec-1s and zVad (Figure 4B). These results indicate that deletion of TAB2 promotes RIPK1-dependent apoptosis and necroptosis. Next, we examined whether TAB2 also regulates apoptosis and necroptosis in cardiomyocytes. Indeed, ablation of TAB2 in neonatal cardiomyocytes with an adenoviral vector encoding TAB2 shRNA (Ad-shTAB2) also promoted TNF-α–induced apoptosis and necroptosis (Figure 4, D–F). To exclude possible off-target effects associated with TAB2 deletion, reconstitution of TAB2-deficient cells with an adenoviral vector expressing human TAB2 restored cellular resistance to TNF-α–induced cell death (Supplemental Figure 6, A–C). Moreover, we further showed that TAB2 ablation promoted TNF-α–induced cell death through the death receptor TNFR1 (Supplemental Figure 6D).

Given that TAB3, a close homolog of TAB2, has been shown to play a redundant role along with TAB2 in mediating TAK1 signaling in several cell types (23, 24), we next assessed whether TAB3 regulates apoptotic or necroptotic cell death in cardiomyocytes. Surprisingly, ablation of TAB3 with Ad-shTAB3 had no effects on TNF-α–induced cell death, HMGB1 release, or PARP cleavage (Figure 4, G–I). Therefore, TAB2 but not TAB3 plays an indispensable and nonredundant role in cell survival by suppressing apoptosis and necroptosis.

TAB2 acts as a molecular adaptor in the TAK1 signaling complex (22), and TAK1 has recently been identified as a key regulator of apoptosis and necroptosis (4, 14). To determine whether TAB2 regulates cell death through a TAK1-dependent mechanism, we assessed whether forced activation of TAK1 rescues cell death in TAB2-deficient cells. Neonatal cardiomyocytes were infected with an adenovirus encoding the constitutively active TAK1 mutant (Ad-TAK1ΔN; ref. 41) in the presence or absence of Ad-shTAB2, followed by stimulation with TNF-α. Indeed, TAK1ΔN largely blocked cell death, HMGB1 release, and PARP cleavage induced by TNF-α in Ad-shTAB2–infected cells (Figure 4, J–L). Therefore, TAK1 activation was sufficient to prevent cell death triggered by TAB2 inhibition, suggesting that TAB2 regulates apoptosis and necroptotic signaling via a TAK1-dependent mechanism.

### TAB2 regulates TNF-α–induced apoptosis and necroptosis mainly through an NF-κB–independent mechanism.

Previous studies in nonmyocytes indicate that TAB2 and TAB3 play redundant roles in linking TAK1 to the TNFR1 signaling complex to mediate downstream NF-κB activation (23, 24). NF-κB is a transcription factor that drives the expression of prosurvival genes, serving as an important cell death checkpoint in TNFR1 signaling. Here, we assessed whether TAB2 is essential for NF-κB activation in cardiomyocytes and whether TAB2 regulates cell death through an NF-κB–dependent mechanism. Surprisingly, in contrast to the redundant roles of TAB2 and TAB3 in nonmyocytes (23, 24), we found that deletion of TAB2 alone was sufficient to block TNF-α–induced NF-κB activation in neonatal cardiomyocytes, with marked inhibition of IκB phosphorylation/degradation and NF-κB transcriptional activity (Supplemental Figure 7, A and B). To further determine whether TAB2 regulates cell death through NF-κB, neonatal cardiomyocytes were infected with an adenovirus encoding the nondegradable IκBα mutant (IκBα-S32/36A; Ad-IκBαM), which completely blocked NF-κB activation (Supplemental Figure 7C), along with Ad-shTAB2 followed by TNF-α stimulation. No significant cell death or HMGB1 release was detected in Ad-IκBαM or Ad-β-galactosidase–infected cells after TNF-α stimulation for 6 hours (Supplemental Figure 7, D–F). In contrast, cell death and HMGB1 release were robustly induced by TNF-α in Ad-shTAB2–infected cells, which were not altered by inhibition of NF-κB with Ad-IκBαM (Supplemental Figure 7, D–F). These data indicate that TAB2 ablation promoted TNF-α–induced rapid cell death response (<6 hours), mainly through an NF-κB–independent mechanism.

Cell death was induced in Ad-IκBαM–infected neonatal cardiomyocytes after prolonged stimulation with TNF-α for 18 hours, which was partially blocked by Nec-1s (Supplemental Figure 7G), suggesting that inhibition of NF-κB promotes a slow cell death response involving both necroptosis and apoptosis, consistent with our recent findings (14). Of note, ablation of TAB2 further increased TNF-α–induced cell death compared with that in Ad-IκBαM–infected cells (Supplemental Figure 7G). Moreover, cell death induced by prolonged TNF-α stimulation in TAB2-deficient neonatal cardiomyocytes was largely, but not completely, abolished by Nec-1s (Supplemental Figure 7G). Therefore, these data suggest that TAB2 deficiency promotes apoptosis and necroptosis through both NF-κB–dependent and –independent mechanisms in the setting of prolonged TNF-α stimulation.

### TAB2 is a key suppressor of RIPK1 kinase activity and RIPK1-dependent apoptosis and necroptosis signaling.

**Figure 5:**
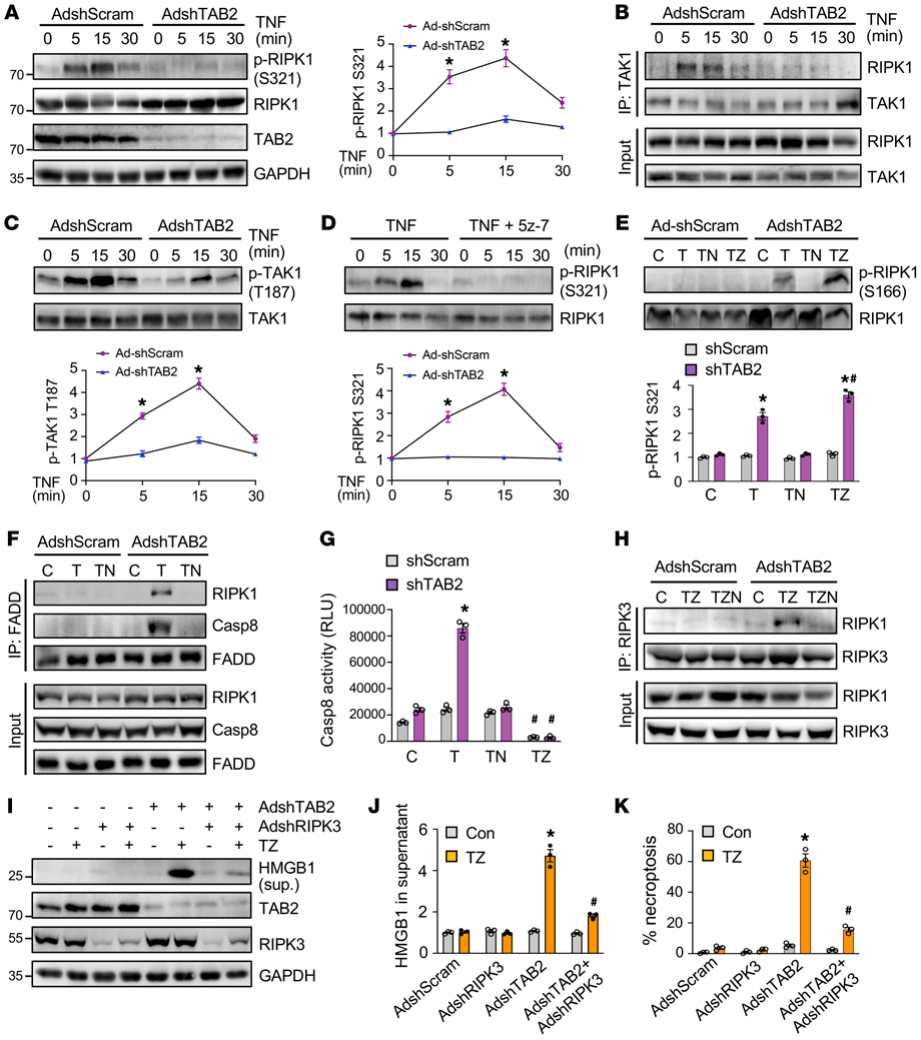
Loss of TAB2 promotes RIPK1-dependent apoptosis and necroptotic signaling. (**A**) Western blotting for the indicated proteins in neonatal cardiomyocytes treated as indicated. *P < 0.05 versus Ad-shTAB2. *n =* 3. (**B**) Western blotting for the indicated proteins after IP with anti-TAK1 from neonatal cardiomyocytes treated as indicated. (**C**) Western blotting and quantification of phospho-TAK1 Thr187 in neonatal cardiomyocytes treated as indicated. *P < 0.05 versus Ad-shTAB2. *n =* 3. (**D**) Western blotting and quantification of phospho-RIPK1 Ser321 in neonatal cardiomyocytes treated as indicated. 5z-7, 5z-7-oxozeaenol. *P < 0.05 versus Ad-shTAB2. *n =* 3. (**E**) Western blotting and quantification of phospho-RIPK1 Ser166 in neonatal cardiomyocytes infected with AdshScram or AdshTAB2 followed by treatment with vehicle control (C) or TNF-α (T) in the presence or absence of necrostatin-1s (N) or zVad-fmk (Z) for 2 hours. *P < 0.05 versus control; ^#^*P* < 0.05 versus shTAB2 T. *n =* 3. (**F**) Western blotting for the indicated proteins after IP with anti-FADD from neonatal cardiomyocytes treated as indicated. (**G**) Caspase-8 activity in neonatal cardiomyocytes. *P < 0.01 versus control; ^#^*P* < 0.05 versus shTAB2 TNF. *n =* 3. (**H**) Western blotting for the indicated proteins after IP with an anti-RIPK3 antibody from neonatal cardiomyocytes treated as indicated. (**I**) Western blotting for the indicated proteins in neonatal cardiomyocytes infected with the indicated adenoviral vectors for 24 hours followed by treatment with TNF-α and zVad-fmk (TZ) for 6 hours. (**J**) Quantification of HMGB1 in cell culture supernatant. *P < 0.05 versus control; ^#^*P* < 0.05 versus Ad-shTAB2 TZ. *n =* 3. (**K**) Necroptosis in cells treated as indicated in **I**. *P < 0.01 versus control; ^#^*P* < 0.05 versus Ad-shTAB2 TZ. *n =* 3. Statistical analysis was performed using 2-way ANOVA with Tukey’s multiple-comparison test.

Our data presented above suggest that TAB2 regulates apoptosis and necroptosis mainly through a RIPK1-dependent mechanism (Figure 4). Next, we investigated the mechanism by which TAB2 regulates RIPK1-dependent cell death signaling in cardiomyocytes. Importantly, RIPK1 underwent a transient phosphorylation at Ser321 upon TNF stimulation, which was completely blocked by TAB2 ablation (Figure 5A). Phosphorylation of RIPK1 by several kinases, including TAK1, has been recently identified as a critical mechanism to suppress RIPK1 kinase activation and RIPK1-dependent cell death (16, 43, 44). We next examined whether TAB2 regulates the interaction between TAK1 and RIP1 by IP. A transient increase in TAK1-RIPK1 interaction was detected upon TNF-α stimulation, which was largely abolished by TAB2 ablation (Figure 5B). Moreover, TAB2 ablation also prevented TNF-α–induced TAK1 phosphorylation at Thr187, a marker for TAK1 kinase activation (41, 45), suggesting that TAB2 critically regulates TAK1 kinase activity in neonatal cardiomyocytes (Figure 5C). Similar to TAB2 ablation, inhibition of TAK1 with 5z-7-oxozeaenol effectively blocked TNF-α–induced RIPK1 phosphorylation at Ser321 (Figure 5D). Together, these results suggest that TAB2 critically mediates TAK1-dependent RIPK1 phosphorylation at Ser321, which represents an important mechanism to prevent RIPK1 kinase activation. Consistent with these results, TNF-α stimulation promoted RIPK1 phosphorylation at Ser166, an indicator of RIPK1 kinase activation (37), in TAB2-deficient cells, especially in the presence of the caspase inhibitor zVad (Figure 5E). To further determine the mechanism by which TAB2 regulates RIPK1-dependent cell death, we performed IP experiments to determine whether ablation of TAB2 promotes the assembly of RIPK1-mediated apoptosis and necroptosis signaling complexes. Co-IP of both RIPK1 and caspase-8 with FADD was detected in TAB2-deficient cells upon simulation with TNF-α, indicating the induction of RIPK1-FADD-caspase-8 apoptotic complex (Figure 5F). Similar results were obtained when co-IP was performed using an anti-RIPK1 antibody (unpublished observations). However, no interaction of FADD with either RIPK1 or caspase-8 was detected in Ad-shScram–infected neonatal cardiomyocytes treated with vehicle control, TNF-α, or TNF-α plus Nec-1s (Figure 5F). Inhibition of RIPK1 with Nec-1s efficiently blocked the RIPK1-FADD-caspase-8 interaction in TAB2-deficient cells, indicating that RIPK1 kinase activity is required for the apoptotic complex formation (Figure 5F). Moreover, TNF-α also induced a marked increase in caspase-8 activity in TAB2-deficient cells but not in control cells, which was blocked by cotreatment with Nec-1s or zVad (Figure 5G). These results indicate that TAB2 negatively regulates RIPK1 kinase activation and its interaction with FADD and caspase-8. Thus, ablation of TAB2 promotes apoptosis signaling and caspase activation in a manner dependent on RIPK1 kinase activity.

The kinase activity of RIPK1 is also essential for the RIPK1-RIPK3 necrosome formation and necroptotic cell death (20, 46). To determine the molecular mechanism by which TAB2 regulates necroptosis signaling, we assessed whether TAB2 ablation promotes the RIPK1-RIPK3 necrosome formation through RIPK1 kinase activation. A marked increase in RIPK1-RIPK3 interaction was detected in TAB2-deficient cardiomyocytes after stimulation with TNF-α and zVad (Figure 5H). No RIPK1-RIPK3 interaction was detected in control cardiomyocytes under the same condition (Figure 5H). The RIPK1-RIPK3 necrosome formation was largely abrogated by Nec-1s, suggesting that RIPK1 kinase activity is required in this process. Moreover, ablation of RIPK3 largely abolished HMGB1 release and necroptotic cell death induced by TNF and zVad in TAB2-deficient cells (Figure 5, I–K). Taken together, these results reveal a new regulatory mechanism by which TAB2 acts as a suppressor of RIPK1 kinase activation though TAK1-mediated phosphorylation, thereby preventing the formation of RIPK1-dependent apoptotic and necroptotic signaling complexes.

### Genetic inactivation of RIPK1 rescues TAB2 deficiency–induced cardiomyopathy in vivo.

**Figure 6:**
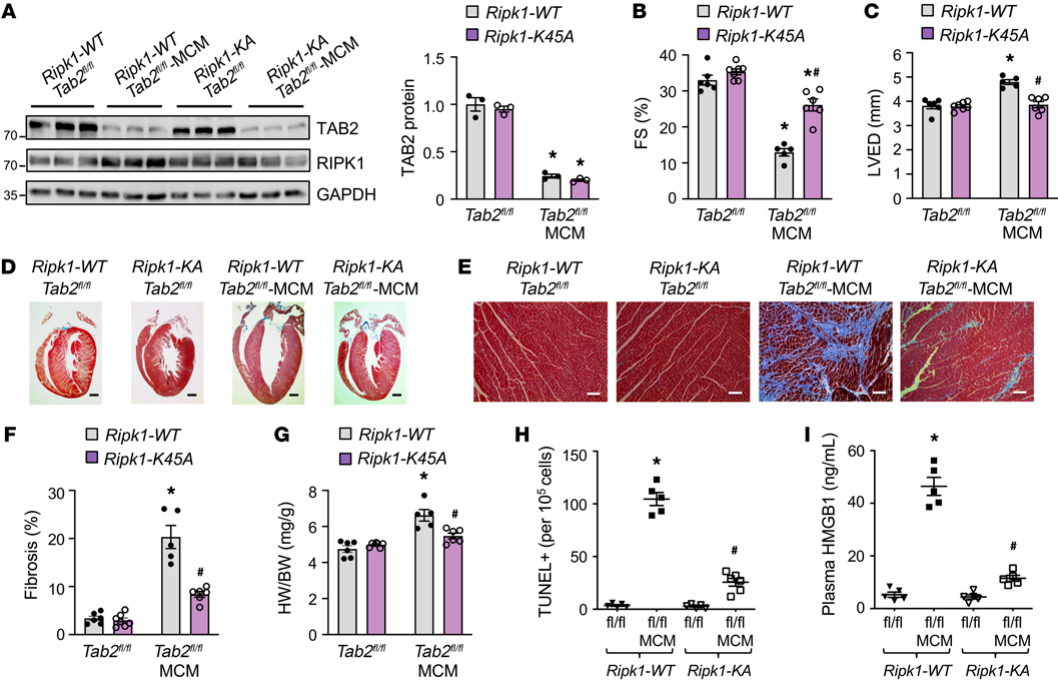
Genetic inactivation of RIPK1 rescues pathological cardiac remodeling and dysfunction in TAB2-deficient mice. (**A**) Western blot and quantification of cardiac TAB2 expression from mice of the indicated genotypes 2 weeks after tamoxifen treatment. *P < 0.05 versus *Tab2^fl/fl^*. *n =* 3. (**B** and **C**) FS and LVED measured by echocardiography from mice of the indicated genotypes 2 weeks after tamoxifen treatment. *P < 0.01 versus *Tab2^fl/fl^*; ^#^*P* < 0.05 versus *Ripk1-WT Tab2^fl/fl^-*MCM. *n =* 5–7. (**D** and **E**) Low- and high-magnification images of Masson’s trichrome–stained cardiac sections from mice indicated in **A**–**C**. Scale bars: 1 mm in **D**; 50 μm in **E**. (**F**) Myocardial fibrosis quantified by MetaMorph software. *P < 0.01 versus *Tab2^fl/fl^*; ^#^*P* < 0.05 versus *Ripk1-WT Tab2^fl/fl^*-MCM. *n =* 5–7. (**G**) Heart weight to body weight ratio (HW/BW) in mice of the indicated genotypes. *P < 0.05 versus *Tab2^fl/fl^*; ^#^*P* < 0.05 versus *Ripk1-WT Tab2^fl/fl^*-MCM. *n =* 5–7. (**H**) TUNEL-positive myocytes in cardiac sections from mice of the indicated genotypes. *P < 0.05 versus *Tab2^fl/fl^*; ^#^*P* < 0.05 versus *Ripk1-WT Tab2^fl/fl^*-MCM. *n =* 5–7. (**I**) Plasma HMGB1 levels from mice of the indicated genotypes. *P < 0.01 versus *Tab2^fl/fl^*; ^#^*P* < 0.05 versus *Ripk1-WT Tab2^fl/fl^*-MCM. *n =* 5–7. Statistical analysis was performed using 2-way ANOVA with Tukey’s multiple-comparison test.

Based on the data presented above, we hypothesize that TAB2 deficiency–induced cardiac remodeling and dysfunction is mediated by RIPK1 kinase activation and the induction of RIPK1-dependent cell death. To test this hypothesis in vivo, we assessed whether genetic inactivation of RIPK1 prevents cardiac cell death and pathological remodeling in TAB2-deficient mice. We crossed *Tab2^fl/fl^*-MerCreMer mice with *Ripk1*-*K45A*-knockin mice, which carry a RIPK1 kinase-dead K45A (lysine 45 to alanine) mutation (47). Mice of different genotypes were confirmed by Western blot analysis of RIPK1 and TAB2 expression in the heart 2 weeks after tamoxifen administration (Figure 6A). *Tab2^fl/fl^*-MerCreMer *Ripk1*-*WT* mice again developed severe contractile dysfunction, ventricular dilation, fibrosis, and hypertrophy after tamoxifen treatment (Figure 6, B−G). This finding was associated with elevated cardiac cell death as indicated by a marked increase in TUNEL-positive cells and plasma HMGB1 levels (Figure 6, H and I). Strikingly, genetic inactivation of RIPK1 largely reversed these pathological changes in TAB2-deficient mice. Indeed, *Tab2^fl/fl^*-MerCreMer *Ripk1-K45A* mice displayed no overt defects in cardiac function or morphology (Figure 6, B–G), with significantly fewer TUNEL-positive cells or less necrotic HMGB1 release compared with *Tab2^fl/fl^*-MerCreMer *Ripk1-WT* mice (Figure 6, H and I). Therefore, these data suggest that elevated RIPK1 kinase activation plays a key role in the pathogenesis of TAB2 deficiency–induced dilated cardiomyopathy in vivo.

To determine whether ablation of *Ripk3*, a key regulator of necroptosis, rescues the cardiac phenotype in TAB2-deficient mice, we crossed *Tab2^fl/fl^*-MerCreMer mice with *Ripk3^–/–^* mice (48). As expected, cardiac dysfunction, ventricular dilation, and myocardial fibrosis were detected in *Tab2^fl/fl^*-MerCreMer *Ripk3^+/+^* mice after tamoxifen treatment (Supplemental Figure 8). However, these effects were only partially alleviated by ablation of *Ripk3* in *Tab2^fl/fl^*-MerCreMer *Ripk3^–/–^* mice (Supplemental Figure 8). Intriguingly, ablation of *Ripk3* in neonatal cardiomyocytes largely blocked necroptosis but mildly increased apoptosis triggered by TAB2 deletion, suggesting a crosstalk between apoptosis and necroptosis (Supplemental Figure 9). Since inhibition of RIPK1 largely blocks both apoptosis and necroptosis (Figure 4), inactivation of RIPK1 using a *Ripk1-K45A*-knockin model is more effective than genetic ablation of *Ripk3* in rescuing the pathological cardiac phenotype of *Tab2*-deficient mice. Taken together, these data reveal a key role for RIPK1-dependent apoptosis and necroptosis in the pathogenesis of TAB2 deficiency–induced dilated cardiomyopathy. These results also provide genetic evidence validating RIPK1 as a therapeutic target for cardiac remodeling and dysfunction associated with TAB2 deficiency and other pathological conditions with aberrant RIPK1 activation.

## Discussion

**Figure 7:**
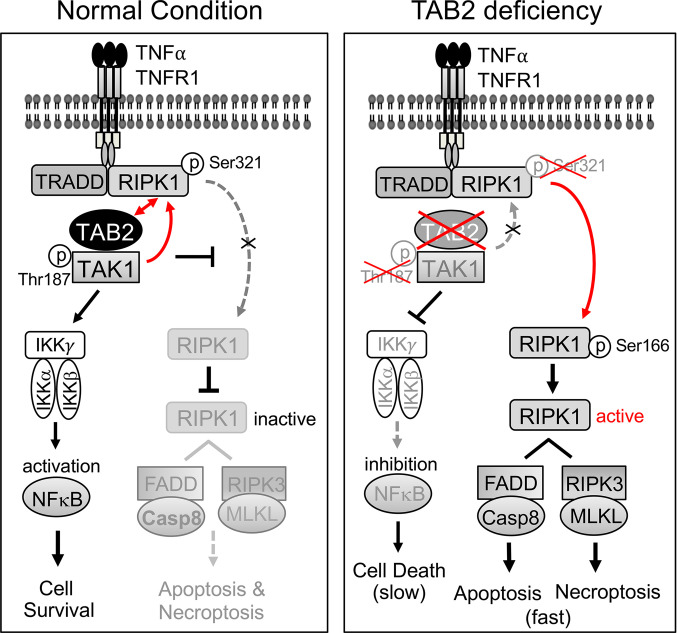
Proposed model: TAB2 acts as a key suppressor of RIPK1 kinase activation and RIPK1-dependent apoptotic and necroptotic signaling. In cardiomyocytes, TAB2 plays an indispensable role in mediating TAK1-RIPK1 interaction, which leads to RIPK1 phosphorylation at Ser 321, preventing RIPK1 kinase activation and RIPK1-dependent apoptosis/necroptosis. TAB2 is also essential for TNFR1-mediated activation of the NF-κB prosurvival pathway. With TAB2 deficiency, TAK1-RIPK1 interaction is disrupted, leading to reduced phospho-RIPK1 at Ser 321, which promotes RIPK1 kinase activation and the induction of RIPK1-FADD-caspase-8 apoptotic complex and RIPK1-RIPK3 necroptotic complex. Moreover, ablation of TAB2 also blocks TAK1 activation, leading to NF-κB inhibition and a slow cell death response distinct from RIPK1-dependent apoptosis/necroptosis.

This study identified an essential role for TAB2 in myocardial survival and homeostasis by suppressing RIPK1 kinase activation and RIPK1-dependent apoptosis and necroptosis. We showed that acute deletion of TAB2 in the adult heart led to dilated cardiomyopathy through the induction of apoptotic and necroptotic cell death, recapitulating the cardiac phenotype in patients with *TAB2* mutations (29–35). Mice with TAB2 deficiency were also predisposed to adverse cardiac remodeling and heart failure after pathological stimulation. Moreover, we provided genetic evidence identifying RIPK1 as a key downstream effector driving cardiac cell death and pathological remodeling in the setting of TAB2 deficiency. Our results support a model that TAB2 acts as a key suppressor of RIPK1 kinase activation and RIPK1-dependent apoptotic and necroptotic signaling by regulating TAK1-RIPK1 interaction and RIPK1 phosphorylation (Figure 7). In cardiomyocytes, TAB2 plays an indispensable and nonredundant role in mediating TAK1-RIPK1 interaction and subsequent RIPK1 phosphorylation at Ser 321, which constitutes a key mechanism preventing RIPK1 kinase activation and RIPK1-dependent apoptosis/necroptosis. TAB2 is also essential for TNFR1-mediated activation of the TAK1/IKK/NF-κB prosurvival pathway (Figure 7). With TAB2 deficiency, TAK1-RIPK1 interaction is disrupted, leading to reduced phospho-RIPK1 at Ser 321, which promotes RIPK1 kinase activation and the induction of RIPK1-FADD-caspase-8 apoptotic complex and RIPK1-RIPK3 necroptotic complex. Moreover, ablation of TAB2 also blocks TAK1 activation, leading to NF-κB inhibition and a slow cell death response distinct from RIPK1-dependent apoptosis/necroptosis (Figure 7). Therefore, TAB2 acts as a nodal regulator of both apoptotic and necroptotic cell death by controlling an early checkpoint via RIPK1 and a late checkpoint at the level of NF-κB.

Patients with TAB2 deficiency often exhibit cardiac dysfunction arising from CHDs, while some display primary cardiomyopathy without CHDs. Our *Tab*2-KO mice developed dilated cardiomyopathy but not CHDs. Of note, here we used αMHC-Cre to drive TAB2 gene deletion during late cardiac development in neonatal mice or MerCreMer to delete TAB2 in the adult heart. None of these *Tab2*-KO models are suitable for studying TAB2 deficiency–induced CHDs. It has been shown that TAB2 is expressed in the embryonic heart tissue of humans and zebrafish, and deletion of TAB2 in zebrafish embryos led to heart abnormalities (29). It will be important to further investigate the role of TAB2 in CHDs using mouse models that ablate TAB2 during early cardiac development. It is also possible that a noncardiomyocyte cell type may contribute to the development of CHDs. Intriguingly, inducible deletion of TAB2 in the adult heart with MerCreMer displayed more severe cardiac remodeling and dysfunction than chronic deletion of TAB2 with αMHC-Cre. We speculate that a compensatory mechanism is induced to maintain myocardial homeostasis in mice with chronic TAB2 deletion. However, in the acute TAB2 deletion model, the compensatory effect did not occur when the gene is acutely deleted in the adult heart, leading to early-onset dilated cardiomyopathy. Differential effects of chronic versus acute gene deletion are also observed in other mouse models, such as in mice with germline versus inducible *Mcu* deletion (49, 50). Moreover, these 2 models have slightly different genetic backgrounds, which may also account for the distinct phenotypic effects (*Tab2^fl/fl^* × MerCreMer is on C57/BL6 background, whereas *Tab2^fl/fl^* × αMHC-Cre has a mixed background of C57/BL6 × 129).

We found that TAB2 protein was markedly downregulated in the heart after pathological stress with pressure overload or MI. The mechanism underlying TAB2 downregulation is unknown but may involve proteasome- or lysosome-mediated protein degradation (51, 52). Importantly, TAB2-deficient mice were predisposed to adverse cardiac remodeling and heart failure after pressure overload or MI, suggesting that TAB2 is also critical for the prevention of pathological cardiac remodeling and heart failure progression. Of note, TAK1 was also downregulated in the heart after prolonged pathological stress (4). We found that overexpression of the active TAK1-ΔN mutant was able to rescue cell death in TAB2-deficient cardiomyocytes (Figure 4K). In our previous study, we generated a transgenic mouse model that drove cardiac-specific expression of TAK1-ΔN and characterized these mice at baseline as well as after pathological stress (53). We observed that transgenic overexpression of TAK1-ΔN in the adult heart inhibited cardiac cell death and protected against pathological remodeling after MI and TAC (53). Together, these results are consistent with the notion that TAB2-TAK1 signaling plays a key role in regulating cardiac cell death during pathological conditions.

Here, we identified a key disease mechanism for TAB2 deficiency–induced dilated cardiomyopathy, which involves aberrant RIPK1 activation and the induction of RIPK1-dependent apoptosis and necroptosis. We speculate this mechanism also underlies the pathogenesis of abnormal cardiac development associated with *TAB2* gene mutation, which warrants further investigation. One of the potentially novel findings of this study is that TAB2 plays an indispensable and nonredundant role in myocardial survival and homeostasis, which overturns the notion that TAB2 plays a redundant role with TAB3 in regulating TAK1 signaling in other systems (23, 24). For example, it has been shown that simultaneous ablation of TAB2 and TAB3, but not either alone, is required to block TAK1-mediated NF-κB and MAPK activation in MEFs and HeLa cells (23, 24). Another observation is the finding that ablation of TAB2 promoted both apoptosis and necroptosis in neonatal cardiomyocytes. In contrast, deletion of TAB3 had no effects on cell death. The role of TAB2 in regulating cell death has been controversial, with differential effects observed in different cell types and cellular contexts (23, 26, 27, 54). Sanjo et al. showed that deletion of TAB2 had no effects in TNF-α–induced cell death in *Tab2^–/–^* MEFs (23). However, *Tab2^–/–^* dermal fibroblasts showed increased sensitivity to TNF-α–induced necroptosis but not apoptosis (26). Instead, deletion of TAB2 promoted LPS-induced apoptosis in hepatocytes (27). Our data revealed an indispensable role for TAB2, rather than a redundant role along with TAB3 (23, 24), in both apoptotic and necroptotic cell death signaling in cardiomyocytes.

Our data support a model whereby TAB2 regulates cell survival and death outcomes through 2 distinct checkpoints. The first checkpoint is controlled by RIPK1 kinase activity; inhibition of RIPK1 prevents it from integrating into the apoptotic complex (RIPK1-FADD-caspase-8) or the necrosome (RIPK1-RIPK3; ref. 55). We found that ablation of TAB2 induces a fast cell death response primarily by promoting RIPK1 kinase activation and the induction of RIPK1-dependent apoptosis and necroptosis, a process independent of NF-κB. The second checkpoint via NF-κB prevents cell death through upregulation of prosurvival genes, such as FLIP and cIAPs (11). We found that TAB2 ablation, through inhibition of NF-κB, induces a slow cell death response involving both apoptosis and necroptosis. Indeed, inhibition of NF-κB promoted cell death after prolonged TNF-α stimulation, which was partially blocked by the RIPK1 inhibitor Nec-1s, consistent with our previous results (14). In contrast to the severe cardiac phenotype in our *Tab2*-deficient mice, cardiac-specific ablation of the major NF-κB subunit *RelA* (NF-κB*-p65*) showed no overt cardiac abnormalities (56, 57). Similarly, mice lacking another NF-κB subunit *Nfkb1* (NF-κB*-p50*) exhibited no detectable cardiac phenotype at baseline (58). These observations suggest that TAB2 regulates myocardial survival and homeostasis mainly through an NF-κB–independent, but RIPK1-dependent mechanism. In support of this notion, our data further showed that genetic inactivation of RIPK1 largely rescued cardiac pathology and cell death induced by TAB2 deficiency in vivo.

Our data identified a key role for TAB2 in suppressing RIPK1 kinase activation, thus preventing apoptotic and necroptotic cell death in the heart. The activation of RIPK1 kinase precedes and is essential for the formation of apoptotic and necroptotic complexes (55). Elevated RIPK1 kinase activity has also recently been implicated in the pathogenesis of inflammatory and degenerative diseases in mice (55, 59, 60). Therefore, the kinase activity of RIPK1 requires active repression to prevent the induction of cell death and inflammation. It has been shown that several protein kinases, including TAK1, IKK, MK2, and TBK1, can phosphorylate RIPK1 at Ser25, Ser321, or Thr190, constituting a critical inhibitory mechanism to suppress RIPK1 kinase activation and subsequent cell death (16, 43, 44). Mechanistically, our data provide new evidence that TAB2 is essential for TAK1-mediated phosphorylation of RIPK1 at Ser321 in neonatal cardiomyocytes, and ablation of TAB2 blocked TAK1-RIPK1 interaction and RIPK1 phosphorylation at Ser321. Moreover, forced activation of TAK1 blocked cell death in TAB2-deficient cells, further suggesting that TAB2 inhibits RIPK1 activation and cell death though TAK1. These results provide a key mechanism by which TAB2 deficiency promotes RIPK1 kinase activation and subsequent integration of RIPK1 into apoptotic or necroptotic signaling complexes. Moreover, ablation of TAB2 also disrupted RIPK1-TAK1 interaction, which may facilitate the dissociation RIPK1 from complex I and its integration into the cell death–inducing complexes. TAB2 functions as an adaptor protein in the TAK1 signaling complex, which mediates TAK1 kinase activation but itself does not bear kinase activity. We observed that overexpression of TAB2 did not promote RIPK1 phosphorylation at Ser321, nor did this inhibit cardiomyocyte necroptosis in vitro, in contrast to overexpression of the constitutively active TAK1-ΔN mutant, which greatly induced RIPK1 phosphorylation at Ser321 and inhibited necroptosis (unpublished observations). Collectively, these results support a model whereby deletion of TAB2 promotes apoptosis/necroptosis by blocking TAK1 activation and subsequent RIPK1 phosphorylation at Ser321 in cardiomyocytes. In contrast to our findings, TAK1 hyperactivation, instead of inactivation, was observed in *Tab2^–/–^* fibroblasts, which was proposed to sensitize cells to necroptosis (26). This discrepancy is likely caused by different cell types used but warrants further investigation.

Our results provide evidence that targeting RIPK1-dependent apoptosis and necroptosis represents a promising therapeutic strategy for TAB2 deficiency. Indeed, genetic inactivation of RIPK1 using a *Ripk1*-K45A-knockin model effectively rescued cardiac dysfunction and remodeling associated with TAB2 deficiency. In contrast, genetic deletion of *Ripk3* only partially attenuated cardiac pathology and dysfunction in TAB2-deficient mice. Consistent with these findings, *Ripk3* deletion largely blocked necroptosis but mildly increased apoptosis in the TAB2-deleted cardiomyocytes, whereas inhibition of RIPK1 kinase activity with Nec-1s inhibited both apoptosis and necroptosis. Recent studies suggest that RIPK1 also has kinase-independent scaffolding functions. The scaffolding function of RIPK1 mediates prosurvival signaling in contrast to the kinase activity of RIPK1, which mediates apoptosis and necroptosis. The prosurvival role of the RIPK1 scaffold is illustrated by the neonatal lethality of RIPK1-KO mice, showing enhanced apoptosis and necroptosis (61, 62). In contrast, mice carrying RIPK1 kinase-dead knockin mutations (K45A, D138N, K584R) showed no abnormalities at baseline and protected against inflammatory and degenerative diseases in mice (59, 63, 64). Therefore, RIPK1 kinase activity, but not its prosurvival scaffolding function, should be targeted to avoid cytotoxic adverse effects. No major adverse effects would be expected for RIPK1 kinase inhibitors if the scaffolding function or expression levels are not affected. Of note, RIPK1 inhibitors are now in clinical trials for the treatment of several human diseases, some of which have successfully passed through phase I clinical studies (46, 55).

In summary, we provide in vitro and in vivo evidence that TAB2 plays an indispensable role in the maintenance of myocardial homeostasis and the prevention of pathological remodeling by suppressing both apoptosis and necroptosis. Mechanistically, TAB2 acts as a key suppressor of RIPK1 kinase activity through TAK1-mediated RIPK1 phosphorylation at Ser321, thereby preventing RIPK1 kinase activation and the formation of apoptotic and necroptotic complexes. Our data also demonstrated that aberrant RIPK1 kinase activation is a key effector driving myocardial apoptosis and necroptosis in vivo, which underlies cardiac remodeling and heart failure induced by TAB2 deficiency. These findings strongly suggest that targeting RIPK1-dependent apoptosis and necroptosis may represent a promising therapeutic strategy for dilated cardiomyopathy in patients carrying TAB2 gene mutations as well as other disease conditions with abnormal RIPK1 kinase activation.

## Methods

### Reagents.

Mouse TNF-α was from R&D Systems. 5z-7-oxozeaenol and puromycin dihydrochloride were from Sigma-Aldrich. zVad-fmk was from Abcam. Nec-1s was from Cell Signaling Technology. Propidium iodide and Hoechst 33342 were from Invitrogen. Lentiviral vectors encoding TAB2 shRNA were obtained from Sigma-Aldrich. The following antibodies were used: anti-TAB2 (catalog 3745), anti-TAB3 (catalog 14241), anti-RIP1 (catalog 3493), anti–phospho-RIPK1 at Ser321 (catalog 38662), anti–phospho-RIPK1 at Ser166 (catalog 53286), anti-RIPK3 (catalog 15828), anti-TAK1 (catalog 4505), anti–phospho-TAK1 at Thr187 (catalog 4536), anti–α-tubulin (catalog 3873), anti-HMGB1 (catalog 6893), anti-PARP (catalog 9532), anti–caspase-8 (catalog 4790), anti–caspase-3 (catalog 9662), anti–cleaved caspase-3 (catalog 9661), anti-Bax (catalog 2772), anti–phospho-IκB at Ser32 (catalog 2859), anti–phospho-JNK (catalog 4668), anti-COL1A1 (catalog 91144), and anti–α-smooth muscle actin (catalog 19245) were from Cell Signaling Technology; anti-FADD (catalog sc-6036), anti-RIPK3 (catalog sc-135171), anti-IκBα (catalog sc-371), Bcl-2 (catalog sc-7382), and anti-GAPDH antibodies were from Santa Cruz Biotechnology; anti-FADD (catalog ADI-AAM-212-E) was from Enzo Life Sciences; anti-MLKL (catalog MABC604) was from MilliporeSigma; anti–TNF-α (catalog AF7014) was from Affinity Biosciences; anti-TNFR1 (catalog AF-425-PB) was from R&D Systems; and anti-ANP (catalog PA5-29559) was from Thermo Fisher Scientific.

### Mouse models.

*Tab2^fl/fl^* mice (23) were provided by Jun Ninomiya-Tsuji and backcrossed to a C57Bl/6J background at least 6 times. *Tab2^fl/fl^* mice were crossed with MCM or αMHC-Cre to generate cardiomyocyte-specific *Tab2*-deficient mice. *Ripk1-K45A* kinase-dead knockin mice (47) were provided by Edward S. Mocarski from Emory University (Atlanta, Georgia, US) and GlaxoSmithKline and were crossed with *Tab2^fl/fl^-*MCM mice. *Ripk3^–/–^* mice (48) were provided by Vishva M. Dixit from Genentech and were crossed with *Tab2^fl/fl^-*MCM mice. In some experiments, mice were treated with tamoxifen (1 mg per 20 g body weight, i.p.) for 5 consecutive days. In most experiments, mice of both sexes aged 2 to 3 months were used unless otherwise stated.

### Echocardiography and surgical procedures.

For echocardiography, mice were anesthetized with 2% isoflurane by inhalation and scanning was performed with a VisualSonics Vevo 2100 imaging system as we described previously (4, 65). M-mode ventricular dimensions were averaged from 3 to 5 cycles. FS was calculated using LVES and LVED: FS = [(LVED – LVES)/LVED] × 100 (%).

TAC was performed to produce cardiac pressure overload in mice using a 27-gauge needle as previously described (56). Sham-operated mice underwent the same procedure without aortic constriction. Doppler echocardiography was performed on mice after TAC to ensure equal pressure gradients across the aortic constriction. Pressure gradients (PGs; mm Hg) across the aortic constriction were calculated from the peak blood velocity (*Vmax*) (m/s) (PG = 4 × *Vmax*^2^). The surgical procedure for MI in mice with permanent ligation of the left anterior descending artery has been described previously (53). For both the TAC and MI surgical procedures, mice were anesthetized with inhaled 2% isoflurane (mice were intubated and respirated throughout).

### Histological analysis and immunofluorescence staining.

Mouse hearts were fixed in 10% formalin/PBS and dehydrated for paraffin embedding. Fibrosis was detected with Masson’s trichrome staining on paraffin sections. Blue collagen staining was quantified using MetaMorph 6.1 software as described previously (4). Assessment of TUNEL from paraffin sections was performed with an ApopTag Peroxidase In Situ Apoptosis Detection kit (MilliporeSigma) according to the manufacturer’s instructions or a TMR Red In Situ Death Detection kit (Roche Diagnostics) as described in detail previously (4, 53). Immunofluorescence staining was performed in frozen heart sections. Heart sections were permeabilized for 2 minutes in 0.1% Triton X-100/PBS and incubated for 1 hour at room temperature in blocking buffer (PBS, 5% goat serum, 2% BSA). Primary antibody incubations were performed overnight at 4°C, followed by Alexa Fluor 488 or 568 conjugated secondary antibodies for 1 hour at room temperature, and subsequently for 10 minutes with DAPI to stain nuclei. Immunoreactivity or TUNEL positivity was quantified from at least 20 random fields by fluorescence or light microscopy.

### Measurement of plasma HMGB1 and TNF-α.

Plasma levels of HMGB1 in mice were measured using an ELISA kit from Chondrex, Inc. according to the manufacturer’s instructions (4). Plasma levels of TNF-α were measured using a TNF-α Quantikine ELISA kit from R&D Systems (5). Absorbance at 450 nm (sample) and 630 nm (reference) was measured with a Synergy 2 Multi-Mode Microplate Reader (BioTek).

### Cell culture.

Primary neonatal rat cardiomyocytes were prepared from hearts of 1- to 2-day-old Sprague-Dawley rat pups as previously described (4, 56). For the isolation procedure, neonatal hearts were collected, the atria were removed, and the ventricles were cut up in HBSS prior to enzymatic digestion. The ventricular tissue was subjected to 5 rounds of enzymatic digestion using 0.05% pancreatin (Sigma-Aldrich) and 84 U/mL collagenase (Worthington). Cells were collected by centrifugation at 500*g* for 5 minutes at 4°C and resuspended in M199 medium. After separation from fibroblasts, enriched cardiomyocytes were plated on 1% gelatin-coated 12-well plates for luciferase assays or on 6 cm diameter dishes for all other experiments. Cells were grown in M199 medium supplemented with 2% bovine growth serum (Thermo Fisher Scientific, SH3054103), 100 U/mL penicillin-streptomycin, and 2 mM l-glutamine. Adult mouse ventricular cardiomyocytes and noncardiomyocytes were isolated using a protocol described by Suetomi et al. (66). Briefly, hearts were perfused with a calcium-free buffer and digested with buffer containing 12.5 μM calcium and 62.5 U/mL collagenase II for 10 minutes. Ventricles were manually dissociated using micro-forceps and by passing through a Pasteur pipette. Cells were pelleted by gravity sedimentation. Pellet was resuspended in perfusion buffer, and magnet bead separation was performed to remove the immune cells and endothelial cells; remaining cells were regarded as cardiomyocyte fraction. Supernatant was collected, filtered by 40 μm strainer, and centrifuged; pelleted cells were regarded as the noncardiomyocyte fraction. *Tab2*-deficient MEFs were generated from WT MEFs by lentivirus-mediated gene silencing and puromycin selection for 2 weeks. Cells were grown in DMEM supplemented with 10% FBS, 100 U/mL penicillin, 100 μg/mL streptomycin, and 2 mM glutamine.

### Adenoviral and lentiviral vectors.

Adenoviral vectors expressing TAB2 shRNA, TAB3 shRNA, RIP3 shRNA, or a scrambled shRNA were generated using the BLOCK-iT Adenoviral RNAi Expression System (Invitrogen) according to the manufacturer’s protocol. The sequence for TAB2 shRNA is 5′-CACCAGTCAA CCCAAGGTCTATAT TCGAAAATATAGACCTTGGGTTGACT-3′. The sequence for TAB3 shRNA is 5′-CACCGCATTACAGCCAGCGTCCTTTACG AATAAAGGA CGCTGGCTGTAATG-3′. The sequence for RIP3 shRNA is 5′-CACCGCTGCTGTCTCCAAGGTAAAGCGAACTTTACCTTGGAGACAGCAGC-3′. The sequence for the scrambled shRNA is 5′-CACCGCCTTAGGTTGGTCGAGAAACGAATTTCTC GACCAACCTAAGG-3′. Ad-h-TAB2 was obtained from SignaGen Laboratories. Adβ-gal, AdTAK1-ΔN, and Ad-IκBαM have been described previously (41). Adenoviral infections were performed as described previously at MOI of 10 to 50 PFUs per mL. Lentiviral particles encoding TAB2 shRNA were obtained from Sigma-Aldrich. Cells were harvested 24 hours after infection followed by Western blot analysis, luciferase assay, or cell death assays.

### Cell death analysis.

Cell death was measured using a Cell Meter Apoptotic and Necrotic Detection kit (ATT Bioquest) as previously described (4, 14). Briefly, cells were incubated at 37°C for 30 minutes with Apopxin green or annexin V Alexa Fluor 488 conjugate for detection of phosphatidylserine on the cell surface, propidium iodide or 7-ADD for labeling the nucleus of cells with membrane rupture, and CytoCalcein for labeling live cell cytoplasm. Cell death was then analyzed with an EVOS FL digital fluorescence microscope (AMG) or a FACSCalibur flow cytometer (Becton Dickinson). Cells with chromatin condensation were visualized by Hoechst 33342 (Invitrogen) staining. Cell viability was also assessed using the Muse Count & Viability assay kit (MilliporeSigma). In brief, cells were trypsinized, washed, and incubated with the Muse Count & Viability reagent, and cell viability was quantified on a Muse cell analyzer (MilliporeSigma).

### Western blot analysis.

Cardiac tissue or cultured cells were lysed using RIPA buffer (50 mM Tris pH 7.5, 150 mM NaCl, 1% NP-40, 0.5% sodium deoxycholate, and 0.1% sodium dodecyl sulfate, 2 mM DTT, 2 mM sodium orthovanadate, 1× protease inhibitor cocktail [Roche]). Equal amounts of protein were subjected to SDS-PAGE and transferred to PVDF membranes (EMD Millipore, IPFL00010). Western blotting followed by enhanced chemiluminescence detection was performed as previously described (4, 5). In some experiments, cell culture supernatants were also collected for the detection of HMGB1.

### IP.

IP was performed as previously described (4, 5, 41). Cells were lysed at 4°C in lysis buffer (50 mM Tris-HCl pH 7.5, 150 mM NaCl, 1 mM EDTA, 10 mM NaF, 1 mM sodium vanadate, 0.5% NP-40) containing protease inhibitor cocktail (Roche). Whole cell lysates were precleared by centrifugation at 18,000*g* for 10 minutes and then incubated with the indicated antibodies and protein A/G-PLUS agarose beads (Santa Cruz Biotechnology) overnight at 4°C. The beads were washed extensively with wash buffer (0.3% NP-40 in PBS), and the proteins were resolved on an 8% to 12% SDS-PAGE for subsequent Western blotting.

### Caspase-8 activity assay.

The caspase-8 activity assay was performed using the Caspase-Glo 8 Assay kit from Promega following the manufacturer’s instructions (14). Briefly, 100 μL Caspase-Glo 8 reagent was added to the cell culture medium in a 96-well plate. Contents of wells were gently mixed using a plate shaker at 500 rpm for 30 seconds. Luminescence was measured with a Synergy 2 Multi-Mode Microplate Reader (BioTek).

### NF-κB luciferase activity assays.

The NF-κB luciferase reporter assay was performed as we previously described (57). Cells transduced with adenoviral vectors encoding the NF-κB luciferase reporter were washed in PBS and then resuspended in lysis buffer (100 mM KH_2_PO_4_, pH 7.8, 0.5% NP-40, and 1 mM DTT). Cellular lysates were centrifuged at 3000*g* for 10 minutes at 4°C and supernatants were assayed in the luciferase assay buffer (100 mM Tris-HCl, pH 7.8, 10 mM magnesium acetate, 1 mM EDTA, 1 mM DTT, 2 mM ATP, and 1 mM luciferin). Luminescence was determined with a Synergy 2 Multi-Mode Microplate Reader (BioTek).

### Statistics.

Results are presented as mean ± SEM. Mice were randomly assigned to experimental and control groups. Investigators were blinded to mouse genotypes during the experiments. Statistical analysis was performed using GraphPad Prism 9. Statistical analysis was performed using the Student’s 2-tailed *t* test for comparison between 2 groups. Comparisons between multiple groups were made using 1-way ANOVA with Tukey’s post hoc test. Comparison of multiple groups with multiple conditions was performed using 2-way ANOVA with Tukey’s multiple-comparison test. *P* values less than 0.05 were considered significant.

### Study approval.

All experiments involving animals were approved by the IACUC of the University of Washington, and all studies were carried out in accordance with the approved protocols.

## Author contributions

QL and HY conceived and designed the study. HY, XG, YC, YZ, XM, SH, HH, JL, and RS carried out experiments and data analysis. HY and XG performed most of the in vivo studies, including echocardiography and surgical procedures. YC and XG generated and characterized *Tab2^fl/fl^-*MCM mice and crossed them into the *Ripk1*-K45A-knockin and *Ripk3*-KO genetic backgrounds. QL supervised the study and wrote the manuscript with input from all authors.

## Supplementary Material

Supplemental data

## Version 1 01/06/2022

In-Press Preview

## Version 2 02/15/2022

Electronic publication
