# Supplementary material for: TAB2 deficiency induces dilated cardiomyopathy by promoting RIPK1-dependent apoptosis and necroptosis
Source: J Clin Invest. 2022 Feb 15;132(4):e152297. doi: 10.1172/JCI152297 (PMC8843707; doi:10.1172/JCI152297)
Supplement: Supplemental data [file jci-132-152297-s180.pdf]

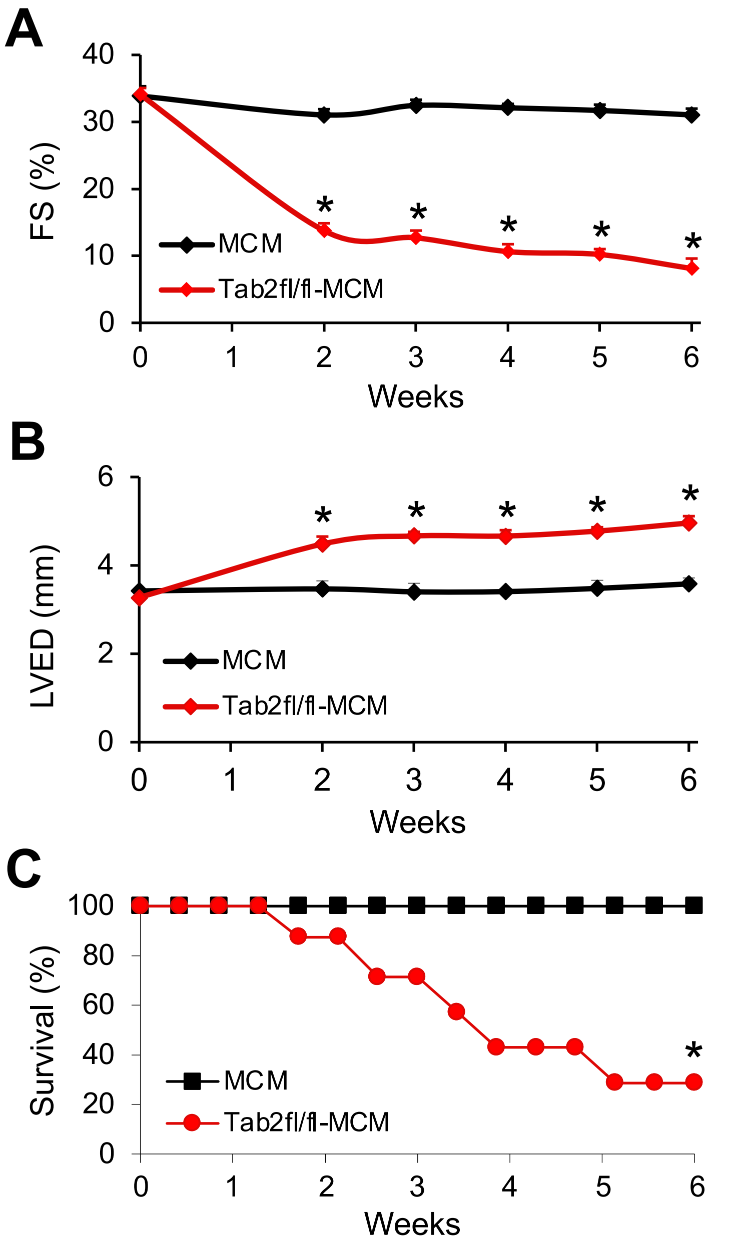

Supplemental Figure 1. Characterization of *Tab2*fl/fl-MerCreMer mice. (**A** and **B**) Echocardiographic assessment of FS in MerCreMer (MCM) and *Tab2*fl/fl-MCM mice at the indicated time points after tamoxifen administration as described in Methods. **P* < 0.05 versus MCM. n = 5-7. Statistical analysis was performed using two-way ANOVA with Tukey's multiple comparisons test. (**C**) Survival rate of the indicated mice treated as in A and B. **P* < 0.05 versus MCM. n = 5-7. Statistical analysis was performed using log-rank test.

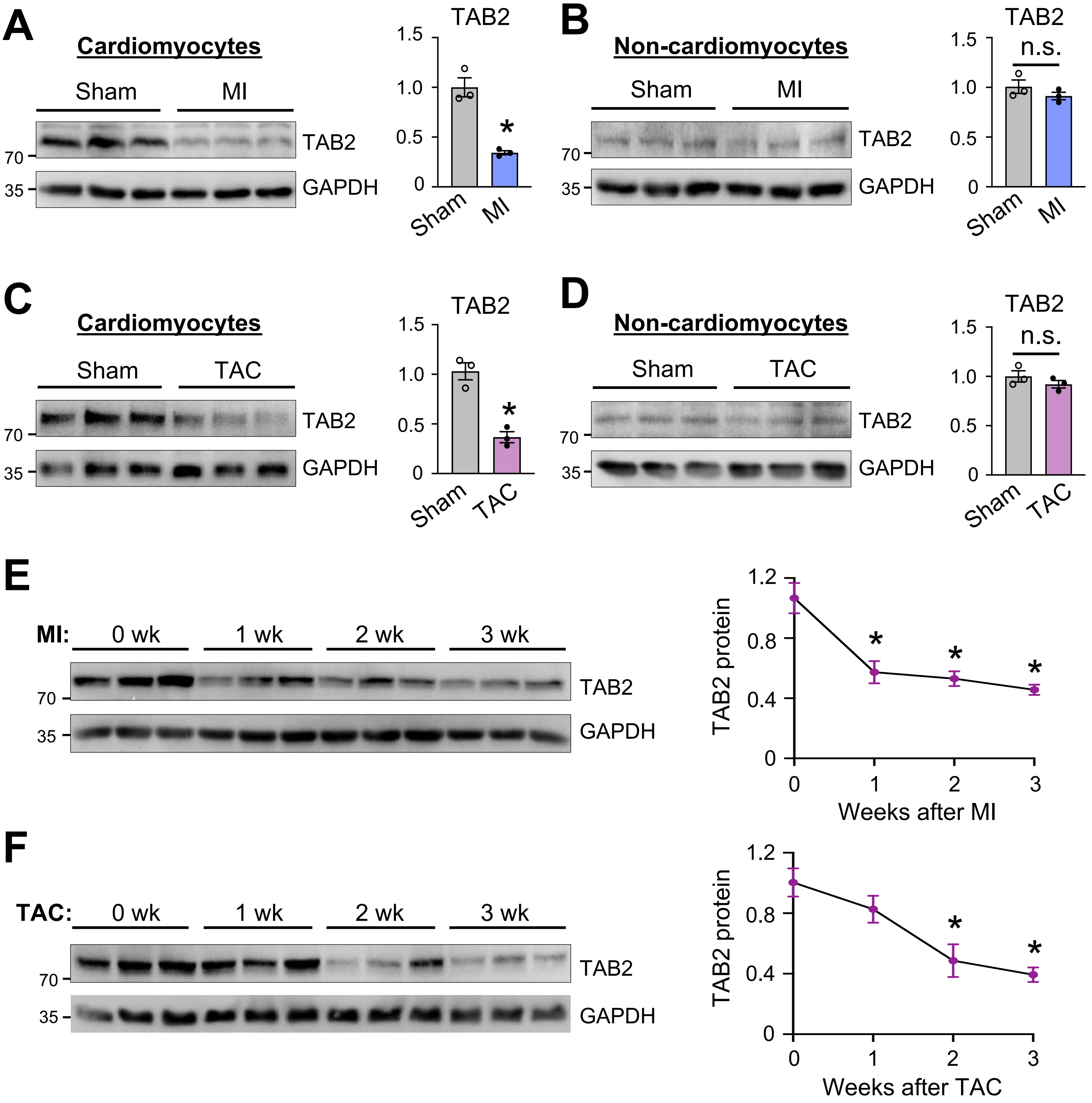

Supplemental Figure 2. TAB2 was downregulated after pathological stress. (**A** and **B**) Western blot and quantification of TAB2 protein level normalized to GAPDH in adult cardiomyocytes (**A**) and non-cardiomyocytes (**B**) from mice subjected to MI or sham surgery for 2 weeks. *P < 0.05 versus Sham. n = 3. (**C** and **D**) Western blot and quantification of TAB2 protein level normalized to GAPDH in cardiomyocytes (**C**) and non-cardiomyocytes (**D**) from mice subjected to TAC (28-gauge) or sham surgery for 2 weeks. *P < 0.05 versus Sham. n = 3. (**E**) Western blot and quantification of myocardial TAB2 protein level normalized to GAPDH in mice subjected to MI for 0-3 weeks. *P < 0.05 versus 0 week. n = 3. (**F**) Western blot and quantification of myocardial TAB2 protein level normalized to GAPDH in mice subjected to TAC (28-gauge) for 0-3 weeks. *P < 0.05 versus 0 week. n = 3. Data were analyzed by Student's t test (A-D) or one-way ANOVA with Tukey's post hoc test (E and F).

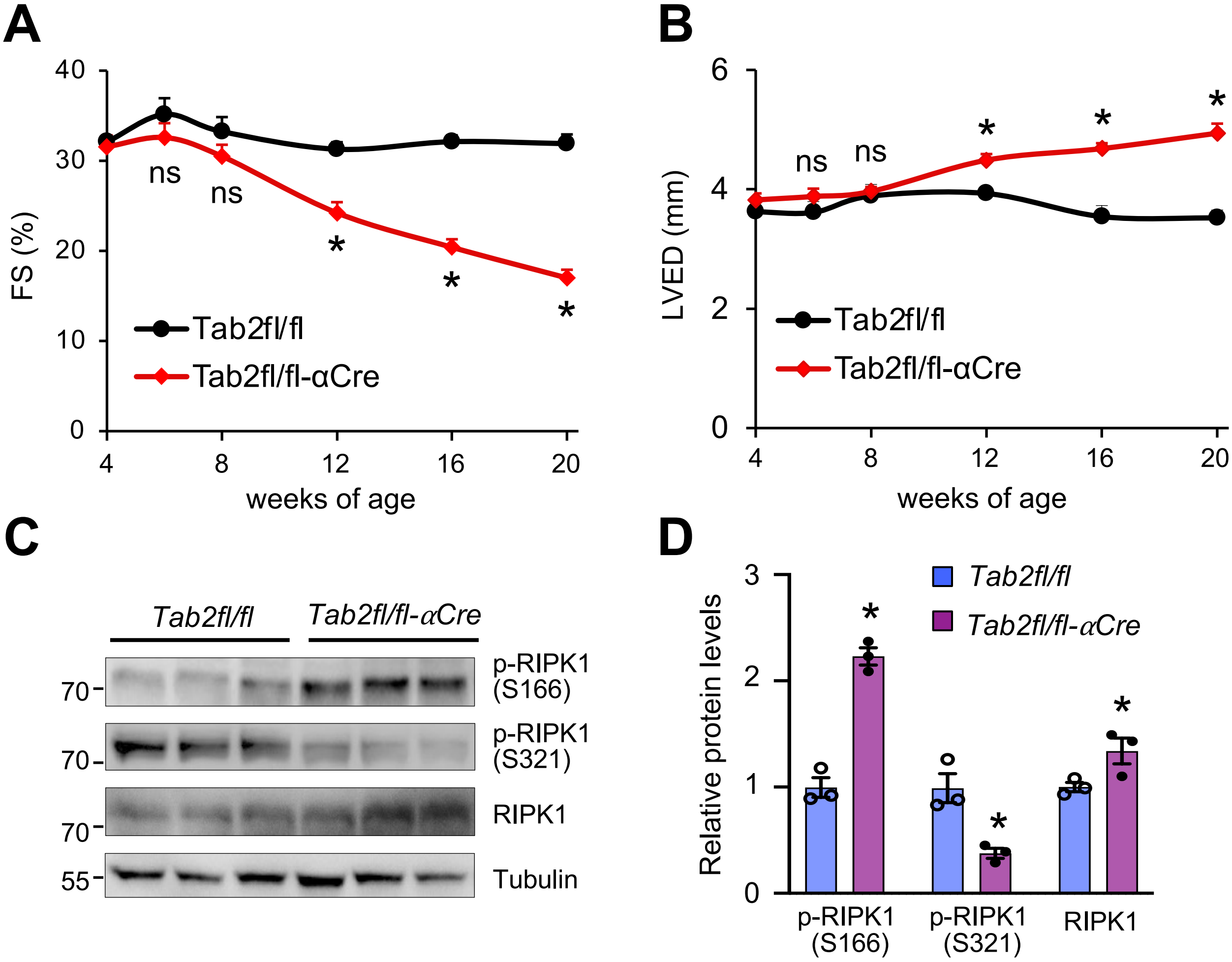

Supplemental Figure 3. Characterization of *Tab2*fl/fl-αMHC-Cre mice. (**A** and **B**) Echocardiographic assessment of FS (fractional shortening) and LVED (left ventricular end-diastolic dimension) in *Tab2fl/fl* and *Tab2fl/fl*-αMHC-Cre mice of the indicated ages. *$P < 0.05$ versus *Tab2fl/fl*. n = 5. ns, non-significant. (**C** and **D**) Western blot and quantification of the indicated proteins normalized to α-Tubulin in cardiac extracts from mice of the indicated genotypes at 20 weeks of age. *$P < 0.05$ versus *Tab2fl/fl*. n = 3. Statistical analysis was performed using two-way ANOVA with Tukey's multiple comparisons test (A and B) or Student's t-test (D).

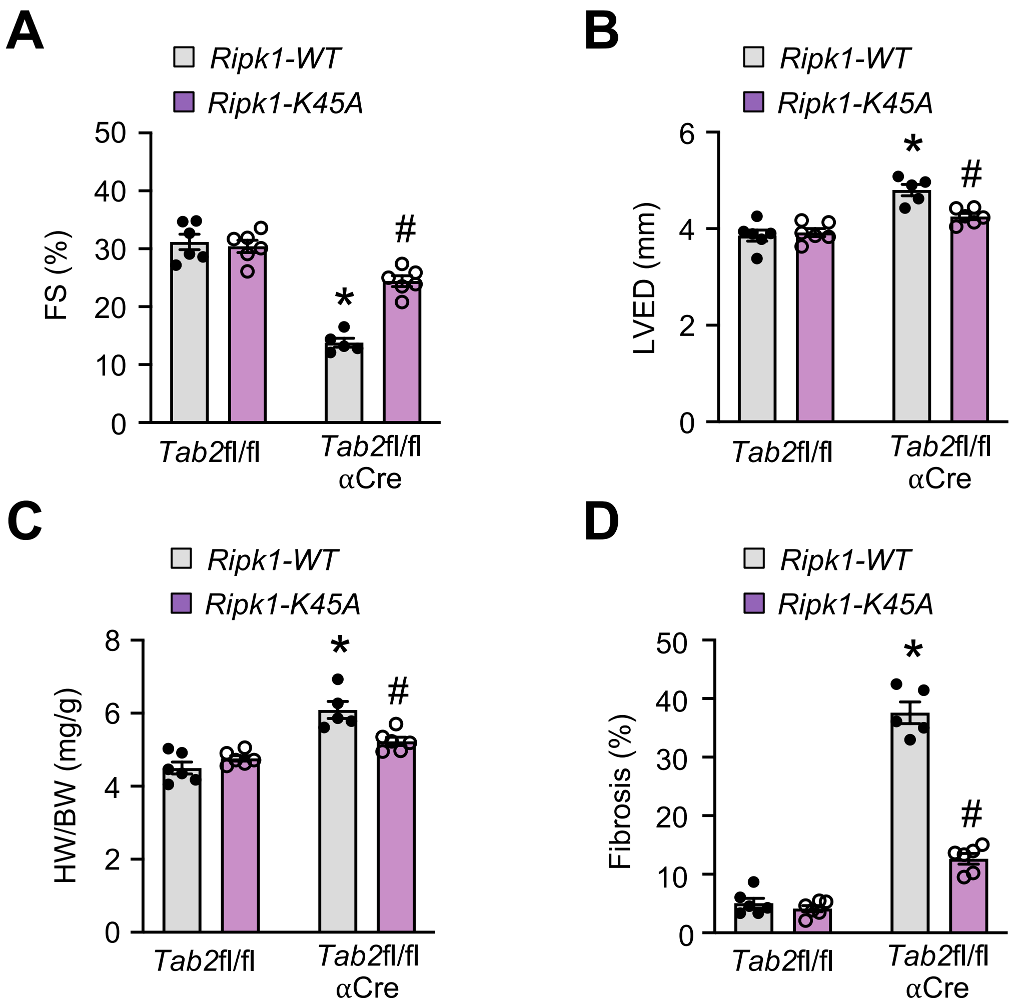

Supplemental Figure 4. Inactivation of RIPK1 rescues cardiac dysfunction and remodeling in *Tab*2fl/fl-αMHC-Cre mice. (**A** and **B**) Echocardiographic assessment of FS and LVED in mice of the indicated genotypes at 4 months of age. *P < 0.05 versus *Tab2*fl/fl. #P < 0.05 versus *Ripk1*-WT;*Tab2*fl/fl-αCre. n = 5-6. (**C**) Heart weight to body weight ratio (HW/BW) in mice of the indicated genotypes at 4 months of age. *P < 0.05 versus *Tab2fl/fl*. #P < 0.05 versus *Ripk1*-WT;*Tab2*fl/fl-αCre. n = 5-6. (**D**) Quantification of myocardial fibrosis. *P < 0.05 versus *Tab2*fl/fl. #P < 0.05 versus *Ripk1*-WT;*Tab2*fl/fl-αCre. n = 5-6. Statistical analysis was performed using 2-way ANOVA with Tukey's post hoc test.

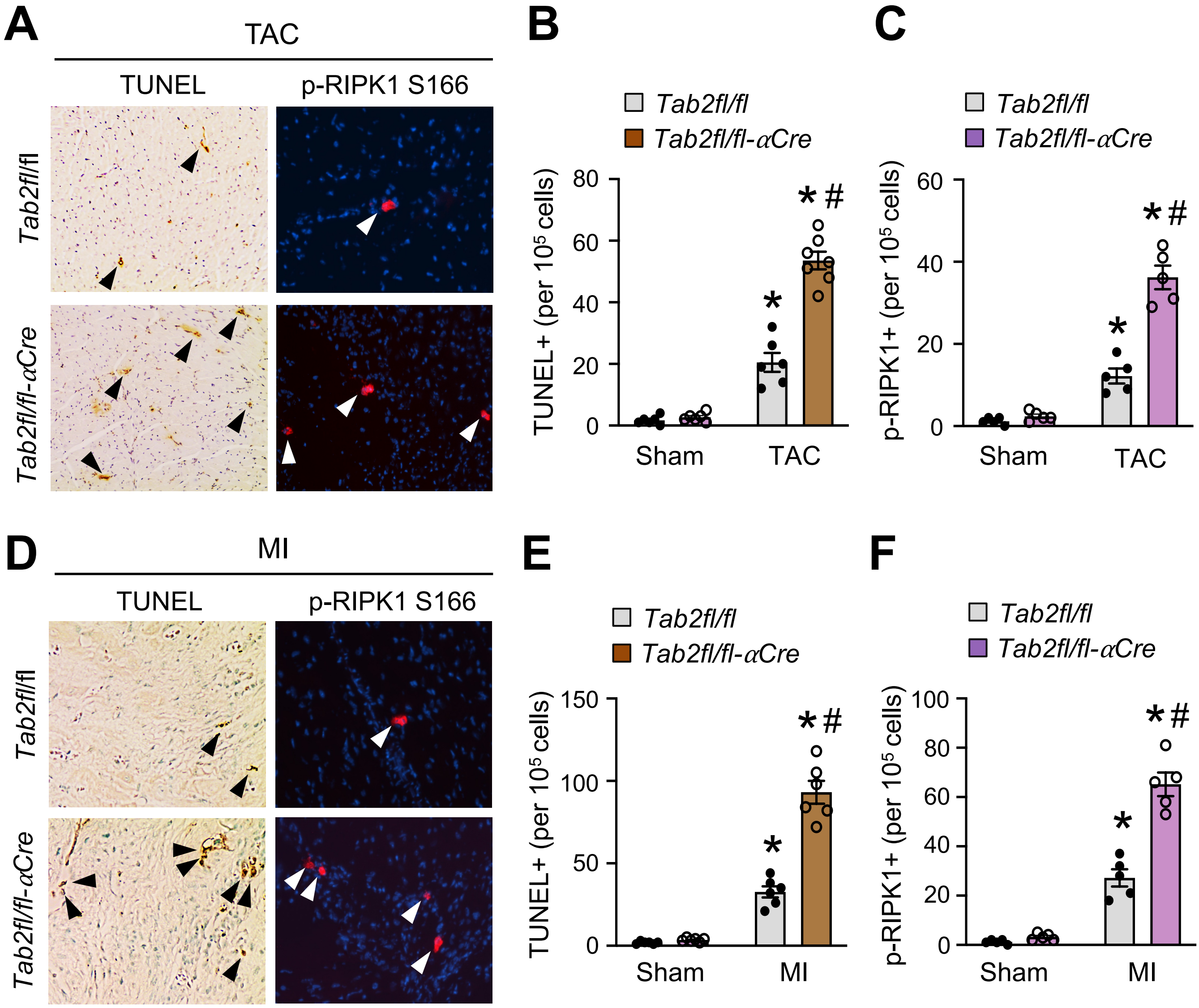

Supplemental Figure 5. Loss of TAB2 promotes myocardial cell death following pathological stress. (**A**) TUNEL assay and immunofluorescence staining for phospho-RIPK1 Ser166 in cardiac sections from mice of the indicated genotypes subjected to TAC for one week. (**B** and **C**) Quantification of TUNEL positive cells and phospho-RIPK1 at Ser166 from samples indicated in A. *$P < 0.05$ versus Sham. #$P < 0.05$ versus *Tab2*fl/fl. n = 5-7. (**D**) TUNEL assay and Immunofluorescence staining for phospho-RIPK1 at Ser166 in cardiac sections from mice of the indicated genotypes subjected to MI for one week. (**E** and **F**) Quantification of TUNEL positive cells and phospho-RIPK1 at Ser166 from samples indicated in D. *$P < 0.05$ versus Sham. #$P < 0.05$ versus *Tab2*fl/fl. n = 5-6. Statistical analysis was performed using two-way ANOVA with Tukey's post hoc test.

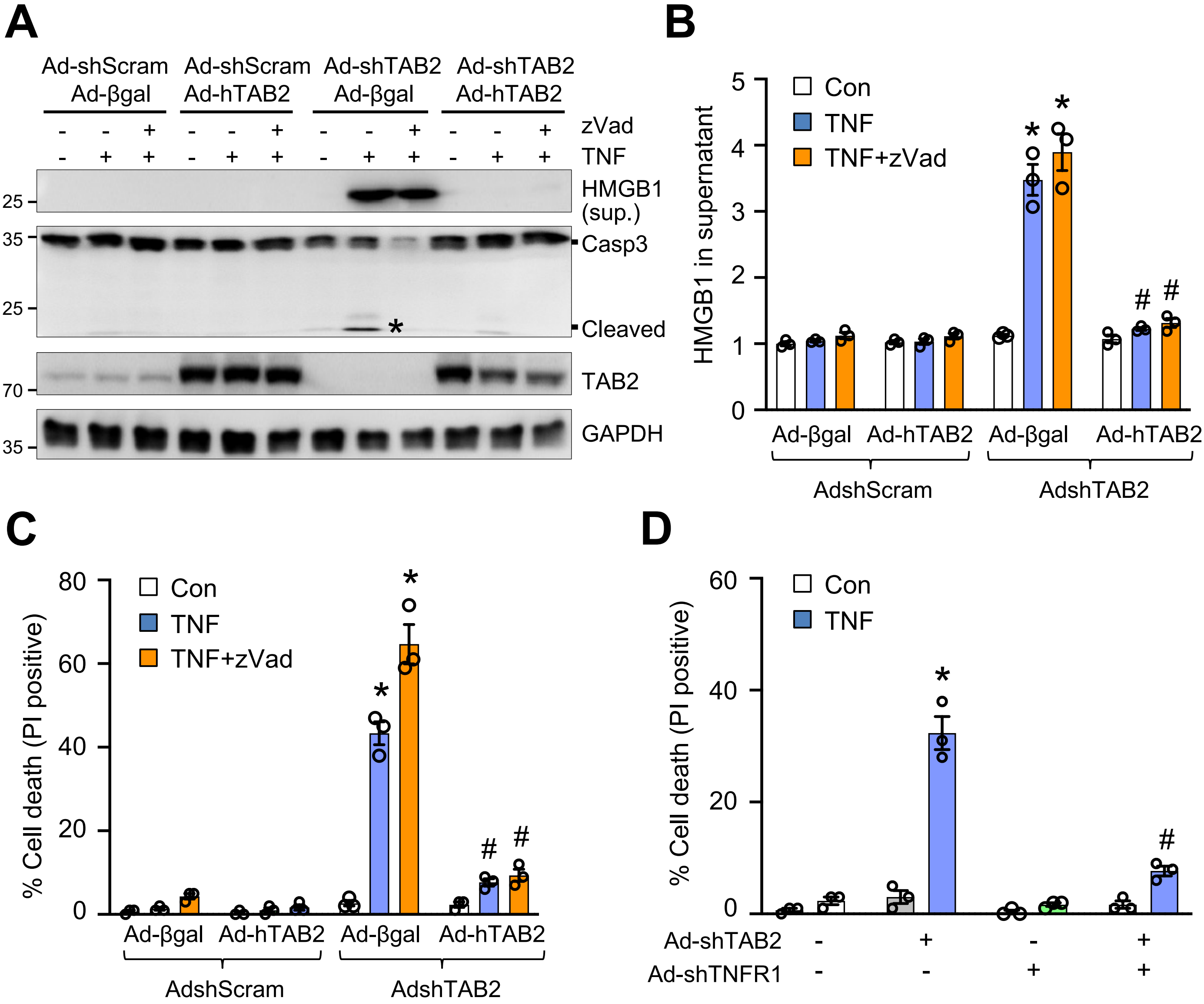

Supplemental Figure 6. Reconstitution with human TAB2 in TAB2-deficient cardiomyocytes restores cellular resistance to apoptosis and necroptosis. (**A**) Western blotting for the indicated proteins in neonatal cardiomyocytes infected with AdshScram or AdshTAB2 for 24 h, followed by infection with Adβ-gal or Ad-hTAB2 for another 24 h and TNFα stimulation in the presence or absence of zVad-fmk for 6 h. hTAB2 indicates human TAB2. *indicates cleaved caspase-3. (**B**) Quantification of HMGB1 in cell culture supernatant as indicated in A. *$P < 0.05$ versus Con; #$P < 0.05$ versus Ad-shTAB2 Ad-βgal in the corresponding group. n = 3. (**C**) Quantification of cell death in cells treated as in A. *$P < 0.01$ versus Con; #$P < 0.05$ versus Ad-shTAB2 Ad-βgal in the corresponding group. n = 3. (**D**) Cell death in neonatal cardiomyocytes infected with the indicated adenoviral vectors for 24 h followed by TNFα stimulation or vehicle control for 6 h. *$P < 0.01$ versus Con; #$P < 0.05$ versus Ad-shTAB2 TNF. n = 3. Statistical analysis was performed using 2-way ANOVA with Tukey's post hoc test.

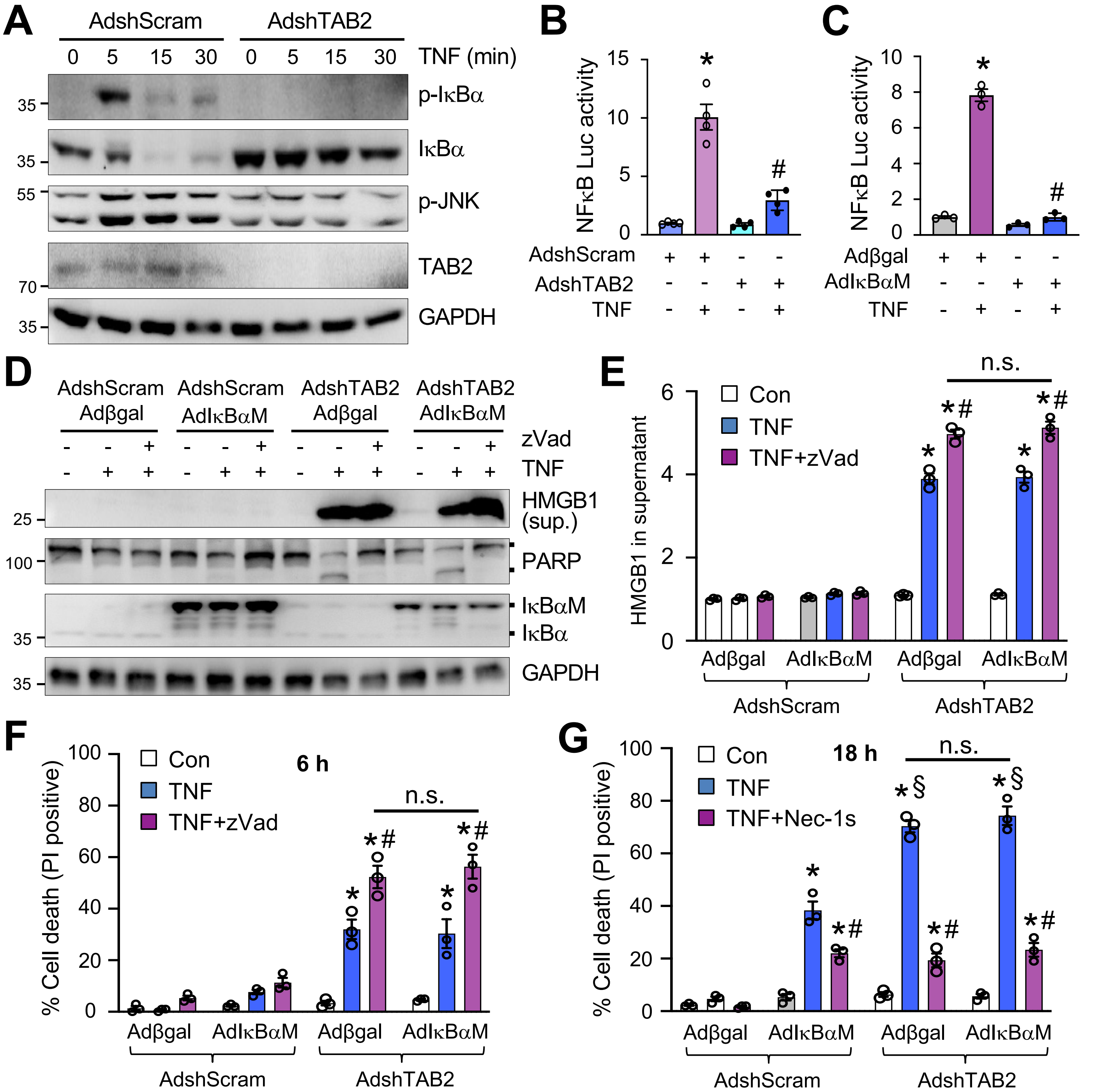

Supplemental Figure 7. TAB2 regulates apoptosis and necroptosis mainly through an NFκB-independent mechanism. (**A**) Western blotting for the indicated proteins in neonatal cardiomyocytes infected with AdshScram or AdshTAB2 for 24 h followed by TNFα stimulation for 0 to 30 min. Data are representative of three independent experiments. (**B** and **C**) NFκB luciferase activity in neonatal cardiomyocytes infected with an adenoviral vector encoding NFκB-luciferase reporter along with AdshTAB2 or Ad-IκBαM (IκBα S32/36A mutant) followed by TNFα stimulation for 12 h. *$P < 0.05$ versus Control; #$P < 0.05$ versus Ad-shScram or Adβgal TNF. n = 3-4. (**D**) Western blots for the indicated proteins in neonatal cardiomyocytes infected with AdshScram or AdshTAB2 along with Ad-IκBαM followed by TNFα stimulation with or without zVad-fmk for 6 h. (**E**) Quantification of HMGB1 in supernatant as indicated in D. (**F** and **G**) Quantification of cell death in neonatal cardiomyocytes treated as indicated for 6 h (F) or 18 h (G). *$P < 0.05$ versus Con; #$P < 0.05$ versus TNF in the corresponding group. §$P < 0.05$ versus AdIκBαM AdshScram TNF. n.s., non-significant. n = 3. Statistical analysis was performed using 2-way ANOVA with Tukey's post hoc test.

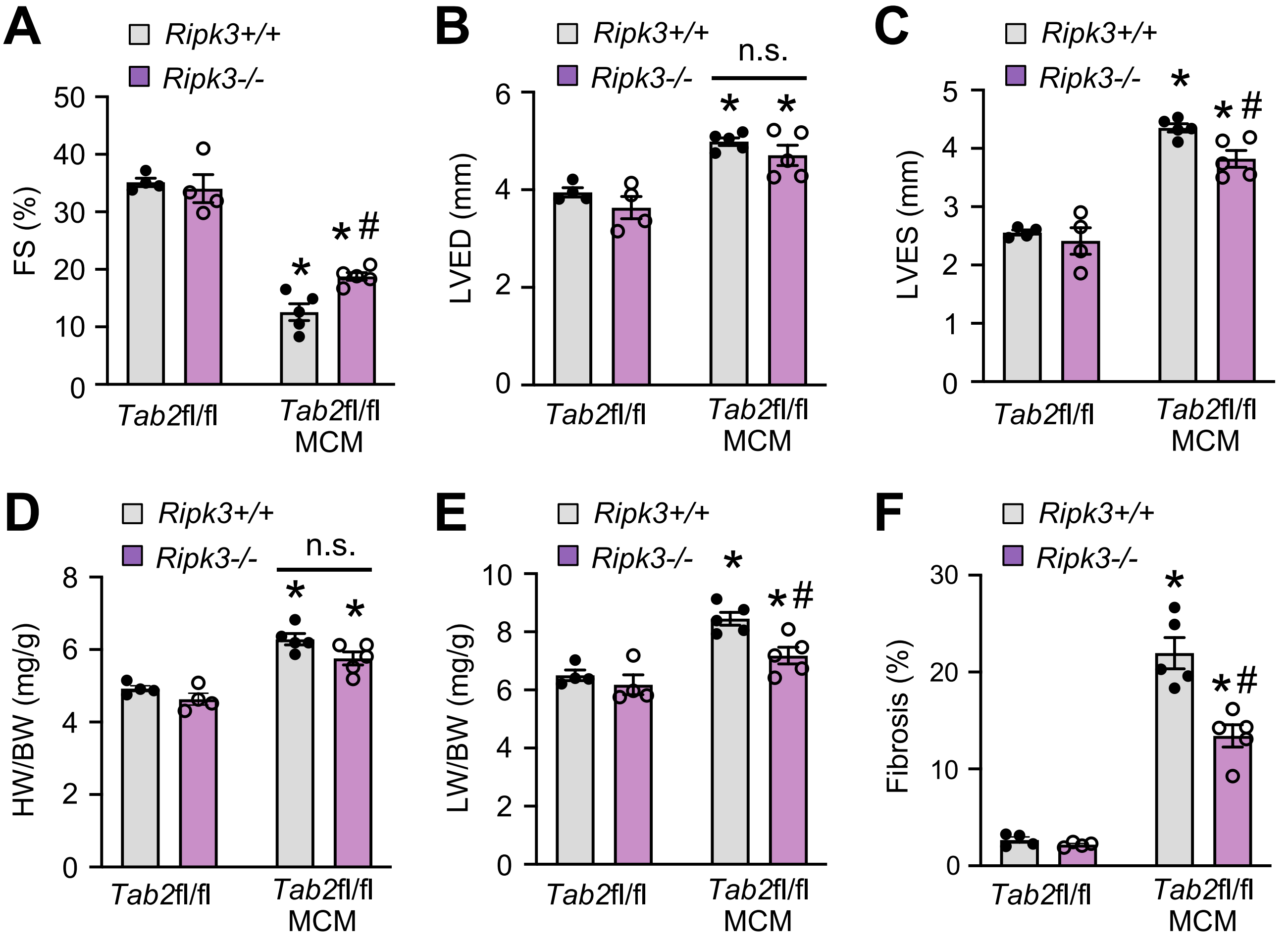

Supplemental Figure 8. Ablation of RIPK3 partially rescues cardiac remodeling and dysfunction in TAB2-deficient mice. (**A**-**C**) Echocardiographic assessment of FS, LVED, and LVES in mice of the indicated genotypes 2 weeks after tamoxifen treatment. *P < 0.05 versus *Tab2fl/fl*. #P < 0.05 versus *Ripk3*+/+;*Tab2fl/fl*-MCM. n.s., non-significant. n = 4-5. (**D**) Heart weight to body weight ratio (HW/BW) in mice of the indicated genotypes. *P < 0.05 versus *Tab2fl/fl*. n.s., non-significant. n = 4-5. (**E**) Lung weight to body weight ratio (LW/BW) in mice of the indicated genotypes. *P < 0.05 versus *Tab2fl/fl*. #P < 0.05 versus *Ripk3*+/+;*Tab2fl/fl*-MCM. n = 4-5. (**F**) Quantification of myocardial fibrosis. *P < 0.05 versus *Tab2fl/fl*. #P < 0.05 versus *Ripk3*+/+;*Tab2*fl/fl-MCM. n = 4-5. Statistical analysis was performed using 2-way ANOVA with Tukey's post hoc test.

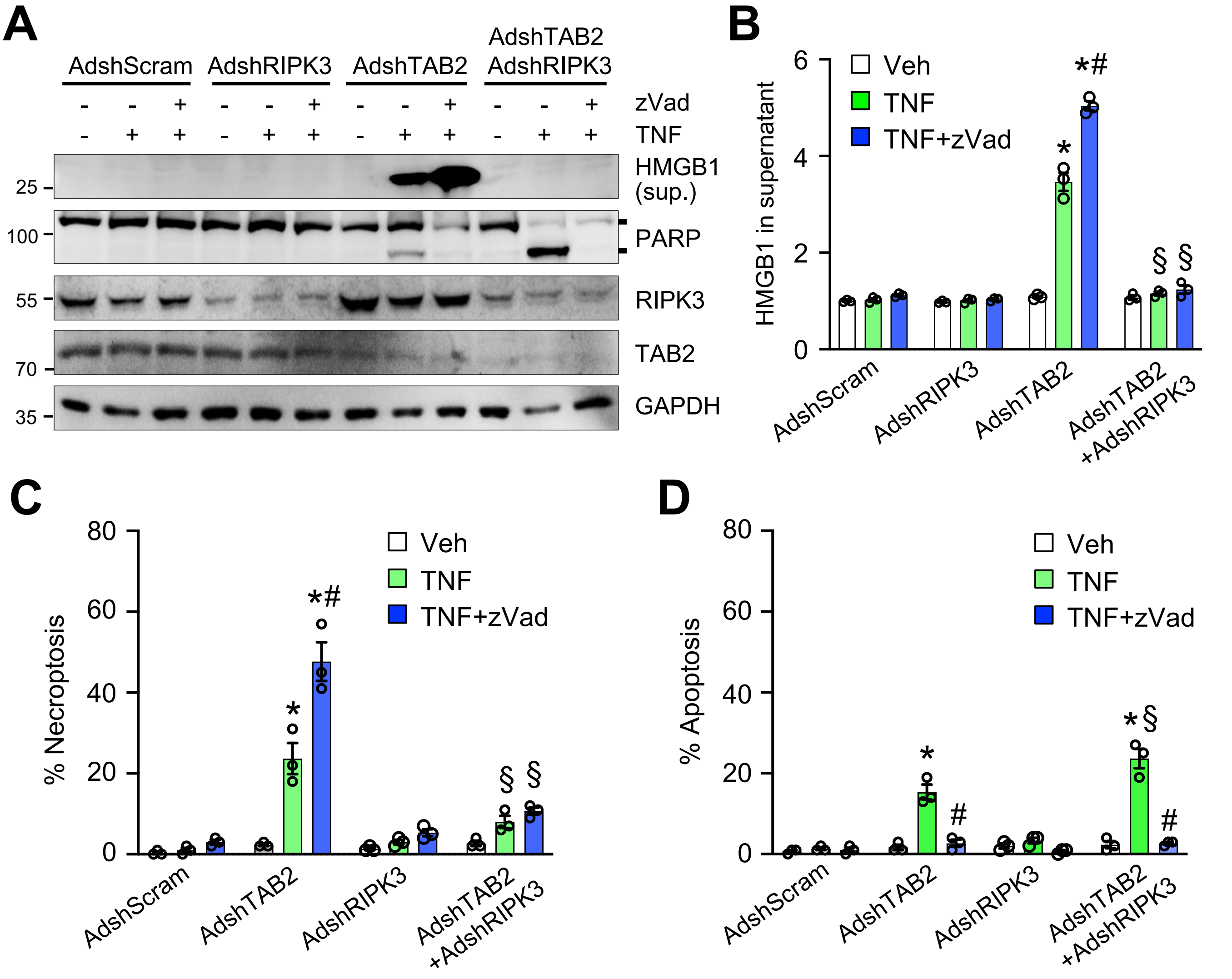

Supplemental Figure 9. Ablation of RIPK3 inhibits necroptosis but mildly increases apoptosis in TAB2-deficient cardiomyocytes. (**A**) Western blotting for the indicated proteins in neonatal cardiomyocytes infected with AdshScram or AdshTAB2 in the presence or absence of an adenovirus encoding RIPK3 shRNA (Ad-shRIPK3) followed by treatment with vehicle control, TNFα, or TNFα+zVad-fmk for 6 h. (**B**) Quantification of HMGB1 in culture supernatant as indicated in A. **P* < 0.05 versus Con; #*P* < 0.05 versus Ad-shTAB2 TNF; §*P* < 0.05 versus Ad-shTAB2 in the corresponding group. n = 3. (**C**) Quantification of necroptosis (PI+/ Annexin V+) from cells treated as in A. **P* < 0.05 versus Con; #*P* < 0.05 versus Ad-shTAB2 TNF; §*P* < 0.05 versus Ad-shTAB2 in the corresponding group. n = 3. (**D**) Quantification of apoptosis (PI-/Annexin V+ with chromatin condensation) from cells treated as in A. **P* < 0.05 versus Con; #*P* < 0.05 versus TNF in the corresponding group; §*P* < 0.05 versus Ad-shTAB2 TNF. n = 3. Statistical analysis was performed using 2-way ANOVA with Tukey's post hoc test.
